# The evolution and functional divergence of *FT*-related genes in controlling flowering time in *Brassica rapa* ssp*. rapa*

**DOI:** 10.1007/s00299-024-03166-2

**Published:** 2024-03-07

**Authors:** Xieshengyang Li, Yan Zheng, Landi Luo, Qian Chen, Tianyu Yang, Ya Yang, Qin Qiao, Xiangxiang Kong, Yongping Yang

**Affiliations:** 1 School of Agriculture, Yunnan University, Kunming, 650091 Yunnan China; 2 Germplasm Bank of Wild Species, Yunnan Key Laboratory for Crop Wild Relatives Omics, Kunming Institute of Botany, Chinese Academy of Sciences, Kunming, 650204 Yunnan China; 3 Institute of Tibetan Plateau Research at Kunming, Kunming Institute of Botany, Chinese Academy of Sciences, Kunming, 650204 Yunnan China; 4 College of Horticulture and Landscape, Yunnan Agricultural University, Kunming, 650201 Yunnan China

**Keywords:** Flowering time, *FT*-related genes, Functional divergence, *Brassica rapa* ssp. *rapa*

## Abstract

**Key message:**

The *BrrFT* paralogues exhibit distinct expression patterns and play different roles in regulating flowering time, and BrrFT4 competes with BrrFT1 and BrrFT2 to interact with BrrFD proteins.

**Abstract:**

Flowering time is an important agricultural trait for *Brassica* crops, and early bolting strongly affects the yield and quality of *Brassica rapa* ssp. *rapa*. *Flowering Locus T* paralogues play an important role in regulating flowering time. In this study, we identified *FT*-related genes in turnip by phylogenetic classification, and four *BrrFT* homoeologs that shared with high identities with *BraFT* genes were isolated. The different gene structures, promoter binding sites, and expression patterns observed indicated that these genes may play different roles in flowering time regulation. Further genetic and biochemical experiments showed that as for FT-like paralogues, BrrFT2 acted as the key floral inducer, and BrrFT1 seems to act as a mild ‘florigen’ protein. However, BrrFT4 acts as a floral repressor and antagonistically regulates flowering time by competing with BrrFT1 and BrrFT2 to bind BrrFD proteins. *BrrFT3* may have experienced loss of function via base shift mutation. Our results revealed the potential roles of *FT*-related genes in flowering time regulation in turnip.

**Supplementary Information:**

The online version contains supplementary material available at 10.1007/s00299-024-03166-2.

## Introduction

*Brassica rapa* plants experienced an extra whole-genome triplication (WGT) event after divergence from *Arabidopsis*, which has affected plant evolution, adaptation, and natural variation (Cheng et al. 2014). The genomic rearrangement and gene evolution initiated by WGT promote the appearance of a variety of *Brassica* plants (Cheng et al. 2014). Cultivated *Brassica* crops exhibit rich developmental and morphological diversity and are generally divided into vegetative and reproductive crops based on the organs consumed (Zhao et al. 2005). Flowering time variations mainly divide *B. rapa* plants into winter-annual and spring-annual habits. Turnip (*Brassica rapa* ssp. *rapa*) is an important crop with edible tubers that belongs to the AA genome of the *Brassica* genus. Turnip is a winter-annual plant that requires vernalization to complete flowering. Sufficient vernalization and subsequent long-day conditions are crucial for the transition from vegetative growth to reproductive growth in turnip (Andres and Coupland 2012; Zhang et al. 2015). Premature bolting severely affects the development of the fleshy root and results in the loss of its commercial value (Zheng et al. 2018, 2021).

WGT also led to changes in gene copy number and divergence in gene functions involved in flowering time regulation. Duplicated genes may retain ancestral functions, may function additively or redundantly, or may develop into sub-, non-, or neofunctionalized genes (Roulin et al. 2013). Previous studies in *B. rapa* showed that *FLOWERING LOCUS C* (*FLC*) paralogues have experienced functional divergence. Four *BraFLC* paralogues have been identified in *B. rapa* crops. Genetic experiments showed that BraFLC2 was the key flowering repressor in *B. rapa* crops, such as turnip (Xiao et al. 2013; Zheng et al. 2018) and Chinese cabbage, whereas BrrFLC5 was expressed at a low level and acted as a weak regulator (Xi et al. 2018). Flowering time is essential for crop reproduction and agricultural production. Hence, studies aimed at understanding the evolutionary divergence of flowering time-related homeologs in *Brassica* crops have high economic relevance.

The main pathways regulating flowering time include the photoperiod, vernalization, autonomous, and age pathways (Jung and Muller 2009). These pathways converge to regulate the floral integrator *FT*-related gene family to influence plant flowering (Takagi et al. 2023). FT is a long-range mobile signal that is produced in leaves and finally transferred to function in the shoot apical meristem (SAM) to promote flowering (Jin et al. 2021; Zhu et al. 2021). *FT* expression is regulated primarily by the photoperiod and vernalization pathways in winter-annual and spring-annual plants, and *FT* transcription is tightly controlled by both positive and negative regulators (Liu et al. 2021). In the leaves, the FLC protein, a strong inhibitor of the vernalization pathway, binds directly to the promoter of the microscopically organized CArG-box structural domain of *FT* to repress *FT* transcription, and prevents the initiation of flowering (Helliwell et al. 2006). The CONSTANS (CO) protein, the key element of the photoperiod pathway, directly activates *FT* gene transcription under long-day conditions (Parcy et al. 1998). At the SAM, the FT protein interacts with the bZIP transcription factor FLOWERING LOCUS D (FD) via 14-3-3 proteins (another class of FT interacting protein), and then promotes the expression of *SUPPRESSOR OF OVEREXPRESSION OF CO1* (*SOC1*) in the apical meristem, directly or indirectly activating downstream gene transcription, such as that of *LEAFY* (*LFY*) and *APETALA 1* (*AP1*), ultimately inducing the formation of the floral meristem and triggering flower development (Abe et al. 2005; Goslin et al. 2017).

*FT*-related genes seem to maintain well-conserved and universal functions across different species. Previous studies have revealed that the presence of corresponding *FT* homologues in plants, such as tomato (*Solanum lycopersicum*) (Lifschitz et al. 2006), citrus unshiu (*Satsuma mandarin*) (Endo et al. 2005), grape (*Vitis vinifera*) (Boss et al. 2006), and poplar (*Populus trichocarpa*) (Hsu et al. 2006), can differentially promote flower development, induce floral transformation, and regulate flowering time. In addition, some studies have reported that *FT* homologues may have different or antagonistic functions, and variations in the segment B external loop, which exists in the 4th exon of *FT*-related genes may explain the functional diversification of *FT* homologues into floral promoters and repressors (Wickland and Hanzawa 2015)**.** For example, in *Beta vulgaris*, *FT2* is important for flowering promotion, but *FT1* suppresses flowering and its expression is downregulated by vernalization (Pin et al. 2010). Some *FT* paralogues from sunflower (*Helianthus annuus*) (Blackman et al. 2010) and tulip (*Tulipa gesneriana*) (Leeggangers et al. 2018) were proven to be involved in repressing flowering, suggesting that functional diversification of *FT*-like genes occurred.

In this study, we isolated 12 *FT*-related genes in turnip, and four were shown to be closely related to *BraFT* homologs annotated in BRAD website by phylogenetic analysis. We investigated the gene structures, expression patterns, and functional characterization of these four *FT-*related genes. Our results revealed the potential roles of *FT*-related genes in flowering time regulation in turnip.

## Materials and methods

### Plant materials and growth conditions

The turnip seeds used in this study were collected from Lhasa, Tibetan Autonomous Region, China. The harvested seeds were sown in Petri dishes containing two pieces of filter paper in the dark at 22 °C until germination. Then, the seedlings were transferred into a greenhouse at 23 °C under long-day conditions to grow one plant per pot (soil: vermiculite = 3:1). The wild-type Col-0 *Arabidopsis* plants used were germinated on MS (Murashige and Skoog) plates, and then transferred into a greenhouse at 23 °C under long-day conditions. For vernalization treatment, the small turnip seedlings were maintained at 5 °C (12 h light:12 h dark) for the indicated days and then transferred to warm and long-day conditions.

### Phenotype of flowering time analysis

The flowering time of *Arabidopsis* was measured as the total number of days to the appearance of the first flower and the total number of rosette leaves when the first flower appeared. For flowering time measurement, at least ten independent plants per genotype were used, and triplicate biological experiments were performed on each genotype (line) at different batches.

### Phylogenetic analysis of FT-related genes

Gene and genome datasets for 8 representative species of *B. rapa* and turnip were downloaded from the Genome Warehouse database (https://ngdc.cncb.ac.cn/gwh/). The *FT*-related gene sequences in *Arabidopsis* were downloaded from TAIR (https://www.arabidopsis.org/). The FT (AT5G59505) amino acid sequence was obtained from TAIR and used as a query to blast FT homologue sequences of different *B. rapa* plants against the genomes of 9 species of *B. rapa*. All FT-related candidate proteins were subjected to multiple sequence alignment using the ClustalW program in MEGA ver.11 software and the website for MAFFT ver. 7 software (https://mafft.cbrc.jp/alignment/software/). The phylogenetic tree was constructed using the neighbour-joining method, bootstrapping with a value of 100, by TreeBeST ver. 0.2.0 software. The subsequent annotation of the tree was completed on the iTOL website (https://itol.embl.de/).

### Gene structure, conserved domain, and motif analysis

The gene structure was predicted on the GSDS2.0 website (Gene Structure Display Server, http://gsds.gao-lab.org/) for genomic DNA sequences. The conserved domains in BrrFT proteins were identified using Tbtools software according to the relevant information from the NCBI Batch CD-search tool (https://www.ncbi.nlm.nih.gov/Structure/bwrpsb/bwrpsb.cgi). The conserved motifs were identified by the online MEME tool (http://meme-suite.org/tools/meme).

### Prediction of promoter cis-elements and transcription factors of *BrrFT* paralogues

The promoter cis-elements were analysed using the website of the PlantCARE database (https://bioinformatics.psb.ugent.be/webtools/plantcare/html/). The potential upstream transcription factors of *BrrFT* promoters were predicted using the website of the PlantPAN2.0 database (http://plantpan2.itps.ncku.edu.tw/TFsearch.php).

### Construction of plant expression vectors and generation of transgenic plants

Four *BrrFT* homologues were cloned using high-quality cDNA from turnip as a template and gene-specific primers according to the complete *Brassica* A genome sequence from the BRAD database (http://brassicadb.cn) and the genome datasets of turnip (https://ngdc.cncb.ac.cn/gwh/). To generate *BrrFT1*, *BrrFT2*, and *BrrFT4* overexpression constructs, the full-length coding region of *BrrFT* genes was inserted downstream of the *cauliflower mosaic virus* (*CaMV*) *35S* promoter at the *SalI* and *EcoRI* sites (Clontech) of the linearized binary plant transformation vector *pRI101-Flag*, generating *35S:BrrFT1-Flag*, *35S:BrrFT2-Flag* and *35S:BrrFT4-lag*, respectively. Then, all the *BrrFT* constructs were transformed into *Agrobacterium tumefaciens* strain EHA105, and Col-0 *Arabidopsis* plants were transformed with *A. tumefaciens* carrying the target gene to generate corresponding transgenic overexpression lines (*BrrFT1-OE*, *BrrFT2-OE*, and *BrrFT4-OE*). Positive transgenic plants were screened with 30 mg/L kanamycin in 1/2 MS solid medium, and further identified by western blotting as previously described (Zheng et al. 2018). Phenotyping was performed with T2 plants. The primers used are listed in Table S1.

### RNA isolation, sequencing, and transcriptome

For RNA-seq analysis, leaves with 2-week-growth plants from turnip without and with 20-day and 40-day vernalization treatment were collected and sequenced. For each sample, three biological replicates were harvested at the same time. Total RNA was extracted using the Eastep® Super Total RNA Extraction Kit (Promega, Madison, WI, USA), the qualified RNA was used to construct an RNA-seq library. The raw data were filtered using SOAPnuke software (v1.4.0, − l 15 − q 0.2 − n 0.1). Then, the clean reads were mapped to reference genome (https://ngdc.cncb.ac.cn/gwh/) by HISAT2 (–very-sensitive –dta). The FPKM (fragments per kilobase of exon per million mapped fragments) method was used to indicate the gene expression levels.

### qRT-PCR analysis

The qRT-PCR analysis was performed as reported previously (Bustin et al. 2009). Different tissues of turnip, including leaves with 60-day old growth, hypocotyls with 60-day old growth, flowers, and floral leaves after 5–6 months of growth, were randomly sampled. The leaves from different developmental stages, including seedlings stage, vegetative stage, floral transition stage before flowering, bolting stage, and flowering stage, were collected under normal growth conditions along with the growth period until turnip flowering. The leaves for analysing vernalization response were obtained from the seedlings which treated with different vernalization treatments and then transferred into greenhouse under normal growth conditions 1 week later. For *Arabidopsis* lines, 10-day old seedlings were harvested for further analysis. These plant tissues and leaves of turnip and *Arabidopsis* were quickly frozen in liquid nitrogen and stored at − 80 °C. Total RNA was extracted using the Eastep® Super Total RNA Extraction Kit (Promega, Madison, WI, USA). Reverse transcription was performed with the NoScript Reverse Transcription System to obtain the first strand of cDNA. qRT-PCR was then carried out using EvaGreen 2 × qPCR MasterMix (ABM) in a StepOnePlus™ Real-Time PCR System (Applied Biosystems) following the manufacturer’s instructions, and the relative expression levels of the genes were calculated using the 2^−ΔΔct^ method. At least three biological replicates and three technical replicates per sample were used in the qRT-PCR analysis. The *TUB2* gene of turnip and *ACTIN2* of *Arabidopsis* were used as controls. The primers used to detect gene transcription levels are listed in Table S1.

### Subcellular localization analysis

Subcellular location assays were performed in the leaves of *Nicotiana benthamiana* as described previously (Sparkes et al. 2006). Briefly, the coding sequences of four *BrrFTs* were cloned and fused to the binary vector *pRI101-GFP* containing *GFP* and *CaMV 35S* promoter. The constructed *35S:BrrFT1-GFP*, *35S:BrrFT2-GFP*, *35S:BrrFT3-GFP*, and *35S:BrrFT4-GFP* expression vectors were transformed into *A. tumefaciens* EHA105 and then infiltrated into tobacco (*N. benthamiana*) leaves. Fluorescence images were obtained using a laser-scanning confocal microscope (Olympus FluoView) after 2–4 days of transformation. The primers used are listed in Table S1.

### LCI assays

LCI assay was performed in the leaves of *N. benthamiana* as described previously (Chen et al. 2008). The full-length cDNAs of *BrrFT1*, *BrrFT2*, *BrrFT4*, *BrrFD1*, and *BrrFD2* were cloned into the *pCAMBIA1300-cLUC* vector (*KpnI/SalI*). Full-length cDNAs of *BrrFD1* and *BrrFD2* were cloned into *pCAMBIA1300-nLUC* (*KpnI/SalI*) vectors, respectively. These constructs were subsequently transformed into *A. tumefaciens* EHA105. Equal volumes of *A. tumefaciens* containing nLUC and cLUC derivative constructs were mixed and coinfiltrated into *N. benthamiana* leaf epidermal cells. The tobacco leaves were sprayed with 100 mM luciferin and incubated in the dark for 10 min at three days after infiltration. The luciferase signal was then detected using an automatic chemiluminescence image analysis system (Tanon 5200 and Lumazone Pylon). At least five leaves were infiltrated and analysed for each experiment. The primers used are listed in Table S1.

## Results

### Evolution and phylogeny of FT-related paralogues in *B. rapa*

**Fig. 1:**
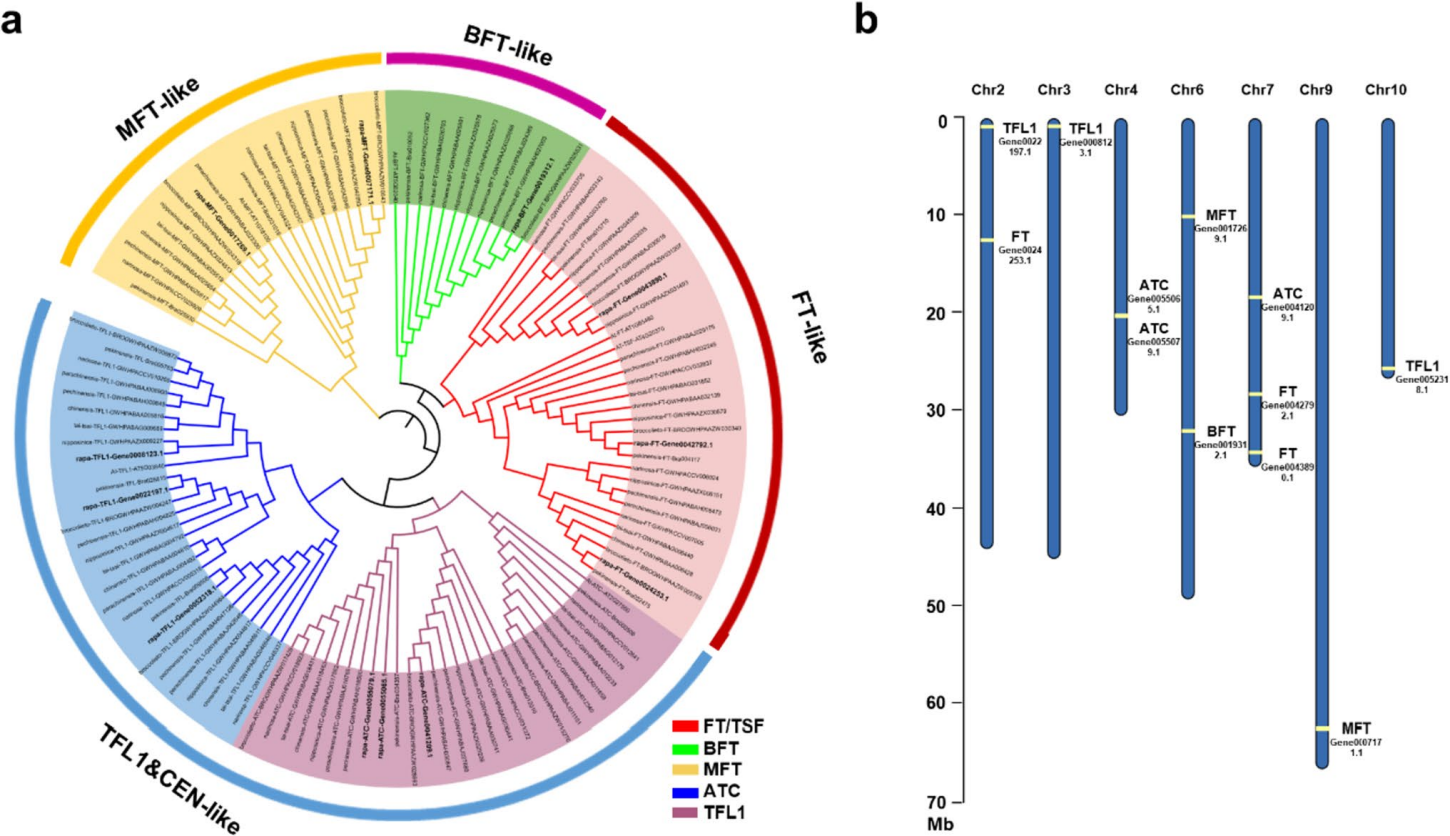
Evolution and phylogeny of FT-related proteins in *B. rapa*. **a** Phylogenetic tree of FT-related proteins in *B. rapa*. The tree was constructed based on full-length protein sequences of FT-related genes using neighbour-joining method in TreeBeST v.0.2.0. FT-related proteins in turnip are shown in bold. Each name contains the name of the plant variety and gene accession number in the Genome Warehouse database. Specifically, pekinensis, *B. rapa* ssp. *pekinensis*; rapa, *B. rapa* ssp. *rapa*; broccolieto, *B. rapa* ssp. *broccolieto*; pechinensis, *B. rapa* ssp. *pechinensis*; parachinensis, *B. rapa* ssp. *parachinensis*; nipposinica, *B. rapa* ssp. *nipposinica*; chinensis, *B. rapa* ssp. *chinensis*; tai-tsai, *B. rapa* ssp. *chinensis* var.*tai-tsai*; narinosa, *B. rapa* ssp. *narinosa*; At, *Arabidopsis thaliana*. **b** Chromosome distribution of turnip *FT*-related genes. The light lines on the chromosome indicate the position of the genes

Reportedly, *B. rapa* species experienced an extra WGT event, and *FT* homoeologs have more than one copy in *B. rapa* (Cheng et al. 2014). A total of 12 *FT*-related genes in turnip were identified using the *Arabidopsis FT* gene as a blast query against turnip genome. To further understand the relationship between the *FT*-related genes from turnip, we constructed a phylogenetic tree using the amino acid sequences of FT homoeologs in 9 representative *B. rapa* species and *Arabidopsis thaliana*. The results showed that *Arabidopsis* FT-related proteins (AtFT/AtTSF, AtTFL1, AtBFT, AtMFT, and AtACT) represented the corresponding main clades, as expected. All *B. rapa* species had at least 12 FT-related proteins, and turnip FT-related proteins could be divided into four main clades: FT-like, MFT-like, BFT-like, and TFL1/CEN-like (Fig. 1a). Phylogenetic analysis showed that three FT-like paralogues of turnip existed in the FT-like clade, three ATC paralogues and three TFL1 paralogues in the TFL1&CEN-like clade, two MFT paralogues in the MFT-like clade and one BFT paralogues in the BFT clade. The 12 FT-related genes were further localized on different chromosomes. Three *TFL1* paralogues were separately localized on chromosomes 2, 3, and 10. Of the *FT* paralogues, one was localized on chromosome 2, and two were localized on chromosome 7 (Fig. 1b). Of the three *ATC* paralogues, two were located on chromosome 4, and one was located on chromosome 7. Of the two *MFT* paralogues, one was on chromosome 6, and the other was on chromosome 9. The *BFT* paralogue was localized on chromosome 6.

### Four BrrFT paralogues belong to a highly conserved plant-specific FT gene family

Four *BraFT* homoeologs have been found to be involved in flowering time in the BRAD (http://www.brassicadb.cn/#/): Bra022475/A02, Bra004117/A07, Bra015710/A07, and Bra010052/A06. Based on the similarity of four BraFT proteins in *B. rapa* Chiffu, we identified four FT homoeologs in turnip that showed the highest similarities with the four BraFT homoeologs, namely Gene0024253.1 (BrrFT1), Gene0042792.1 (BrrFT2), Gene0043890.1 (BrrFT3), and Gene0019312.1 (BrrFT4) (Fig. S1).

**Fig. 2:**
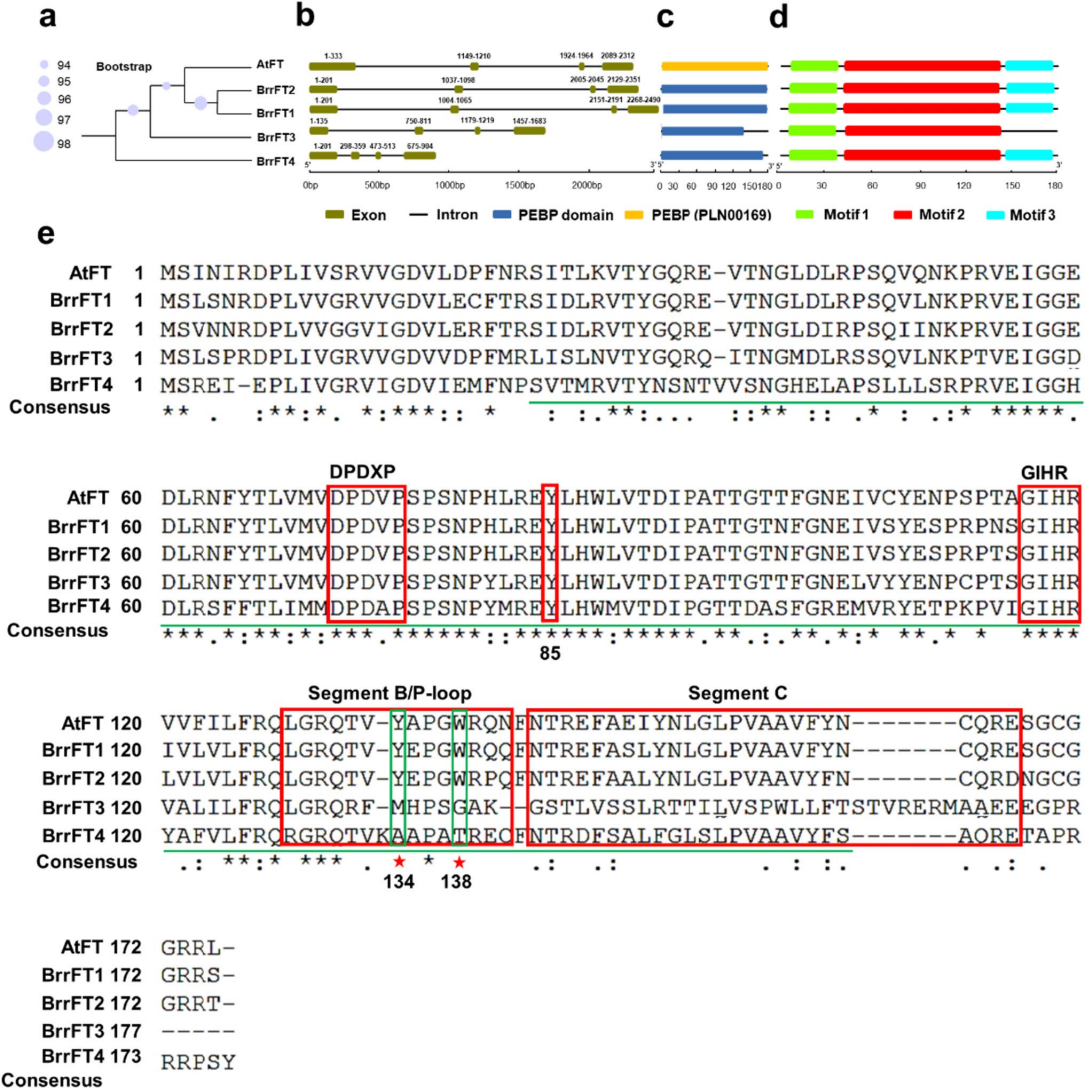
Gene structure analysis and multiple alignments of four BrrFT sequences. **a** The phylogenetic tree of four BrrFT proteins and AtFT proteins using TreeBeST v.0.2.0 software. **b** Gene structure of four *BrrFT* genes and the *AtFT* gene. The lines indicate introns, and the boxes indicate exons. **c** The location of PEBP conserved domain. **d** The motif distribution in four BrrFT proteins determined using the MEME web server. The motifs numbered 1–3 are displayed in different coloured boxes. **e** Multiple sequence alignments of four BrrFT protein sequences with AtFT and AtBFT in *Arabidopsis*. Conserved domains are underlined and boxed

To validate the possible function of four *FT* paralogues in turnip, we isolated the four *BrrFT* homologues from turnip by specific primers designed based on the complete genome sequence of turnip. The amino acid sequences of four turnip BrrFT paralogues were compared with *Arabidopsis* AtFT. The putative turnip BrrFT1 and BrrFT2 were closely related to each other, with 90.29% identity, and to AtFT, with ~ 85.71% and 81.71% identity (Table S2). BrrFT4 showed only ~ 55% identity with BrrFT1, BrrFT2, and AtFT. However, BrrFT3 shared a low percentage of similarity with the ATFT protein, and a single base deletion shift mutation was found in BrrFT3 compared to that of Chinese cabbage (Fig. S2), which indicated that the *BrrFT3* gene may be a non-functionalization type of gene compared to that in Chinese cabbage. The phylogenetic tree also showed that BrrFT1 and BrrFT2 were closely related to the *Arabidopsis* FT (Fig. 2a). The genomic structure of *BrrFT* paralogues varied greatly due to intronic size variations, while the *BrrFT* paralogues contained three conserved intron structures and four exons (Fig. 2b, Table S3). The characteristic PEBP domain was identified in all four BrrFT proteins (Fig. 2c). BrrFT1, BrrFT2, and BrrFT4 shared three similar conserved motif (motif 1, motif 2, and motif 3) compositions, with BrrFT3 having only motif 1 and motif 2 (Fig. 2d), which was due to the single base deletion-related shift mutation.

Further multiple sequence alignments showed that the DPDXP and GIHR regions were conserved in the four BrrFT proteins, and segment B and segment C regions existed in the four BrrFT proteins, while some site mutations existed in BrrFT3 and BrrFT4. Specifically, we found that the tyrosine (Y) at position 85 was conserved among the four BrrFT proteins. However, the tyrosine (Y) at position 134 and tryptophan (W) at position 138, which were located in the segment B region, were conserved in BrrFT1 and BrrFT2 but not in BrrFT3 and BrrFT4 (Fig. 2e). Previous studies found that the conserved amino acids located at positions 85 and 134/138 in the segment B domain determine the variation in the repressor or inducer role of FT homoeologs (Wickland and Hanzawa 2015; Jin et al. 2021). These results indicated that BrrFT1 and BrrFT2 may have conserved functions in promoting flowering, but BrrFT4 may have evolved antagonistic functions in flowering time regulation.

### Subcellular location and spatial expression patterns of four BrrFT paralogues

**Fig. 3:**
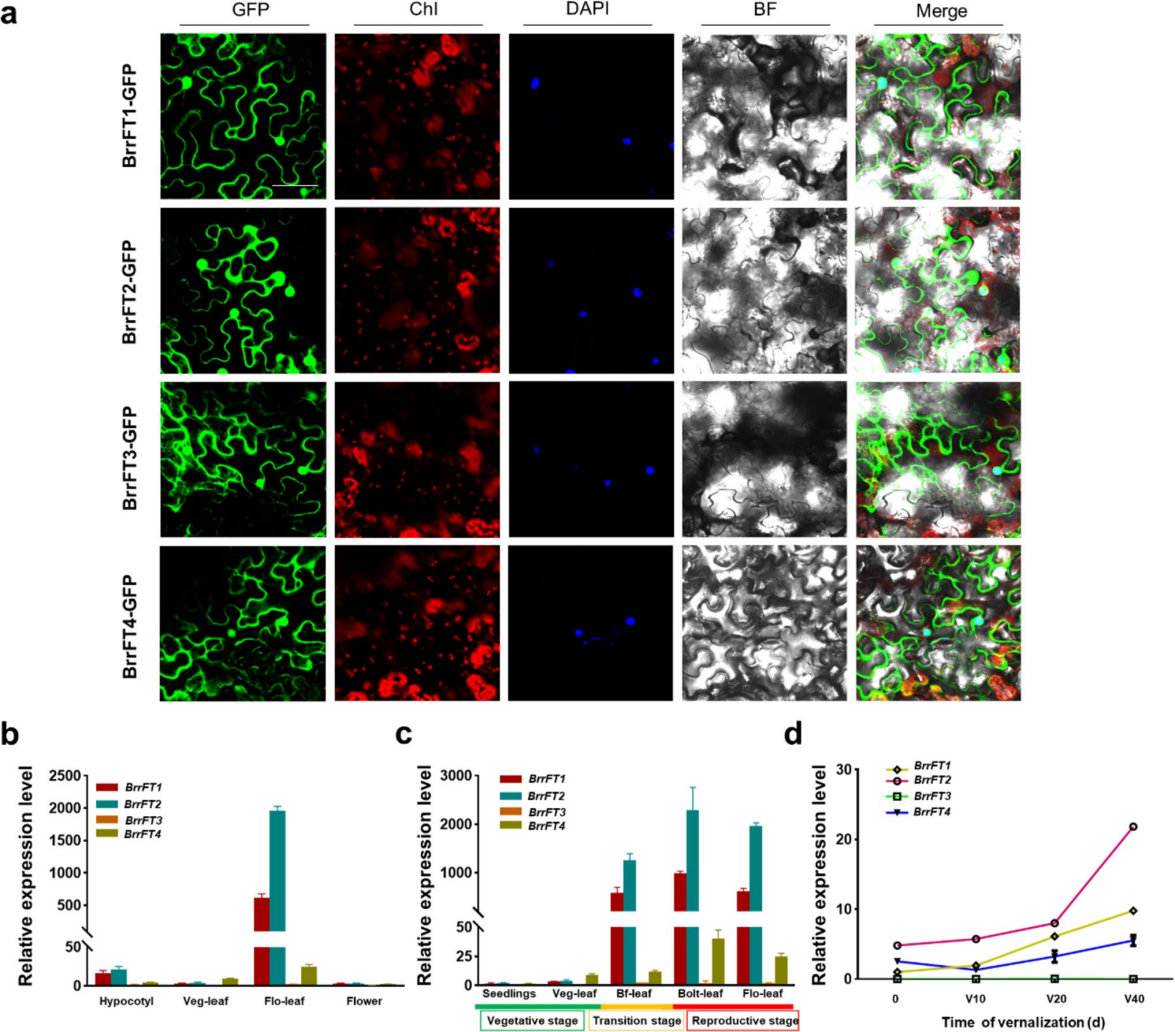
Spatial expression patterns of *BrrFT* homologues in turnip. **a** Subcellular localization of BrrFT homologue fusion proteins in *N. benthamiana* leaf cells. GFP fluorescence was detected in leaves of *N. benthamiana* (bar = 20 µm). **b** Expression level of *BrrFTs* in different tissues of turnip. The hypocotyl, vegetative leaves, floral leaves, and flowers used in detecting transcripts of *BrrFT* homologues were collected at the same time. **c** Relative expression levels of *BrrFT* homologues in leaves of turnip at different developmental stages (seedlings stage, vegetative stage, floral transition stage before flowering, bolting stage, and flowering stage) under long-day conditions. **d** Relative expression patterns of four *BrrFT* homologues with different vernalization treatments. Seedlings were collected one week after vernalization. The expression levels of *BrrFT* paralogues were normalized to that of *TUB2*. Data are mean ± SD, *n* = 3

To investigate the expression locations of the four BrrFT proteins in cells, we generated the *35S:BrrFT1-GFP*, *35S:BrrFT2-GFP*, *35S:BrrFT3-GFP*, and *35S:BrrFT4-GFP* constructs, and detected the subcellular localization of these four BrrFT proteins using the transient expression system in tobacco (*N. benthamiana*) leaves. We found that the four proteins were all expressed in the nucleus and cytoplasm of the cells (Fig. 3a). We further analysed the expression patterns of the four *BrrFT* homologues in different tissues (hypocotyl, vegetative leaf, floral leaf, and flower) of turnip (Fig. 3b). We found that the expression level of the *BrrFT* homologues was highest in floral leaves compared to the low expression levels in other tissues. In particular, *BrrFT1* and *BrrFT2* were expressed at much higher levels than *BrrFT3* and *BrrFT4*, and *BrrFT2* was expressed at the highest level. *BrrFT4* was also expressed at the highest level in floral leaves compared to other tissues but at much lower levels than *BrrFT1* and *BrrFT2*. However, *BrrFT3* exhibited an extremely low expression level across all detected tissues.

Furthermore, we also analysed the transcript levels of *BrrFT* homologues at different developmental stages, including the vegetative growth stage, transition stage, and reproductive growth stage (Fig. 3c). We found that *BrrFT* expression was activated rapidly near the floral transition stage and maintained a high level during the whole blooming stage but was hardly detected in leaves of the vegetative growth stage. *BrrFT1* and *BrrFT2* expression was strongly activated in the transition stage and reproductive stage, and *BrrFT2* was expressed at a higher level than *BrrFT1*. *BrrFT4* was also activated after transformation into the reproductive stage, with much lower expression level compared with that of *BrrFT1* and *BrrFT2*. *BrrFT3* was hardly detected in all developmental stages. These results indicated that *BrrFT1* and *BrrFT2* were positively induced by floral transition, and possibly involved in this process, and *BrrFT4* seemed to also be involved in floral transition. However, *BrrFT3* was hardly detectable in all of the detected tissues and developmental stages. Based on this result combined with its sequence shift mutations, we speculated that *BrrFT3* may be loss-of-function in regulating flowering time.

As a kind of winter-annual plant, turnip requires vernalization to complete flower transition (Zheng et al. 2018). To verify whether *BrrFT* paralogues functioned in the vegetative stages before vernalization or floral stages after vernalization, we determined the expression pattern of the four *BrrFT* paralogues before and after different vernalization treatments (Fig. 3d). The expression levels of *BrrFT1* and *BrrFT2* showed similar expression patterns after vernalization treatments, with expression induced to a significantly high level, and *BrrFT2* was expressed at a relatively high level compared to *BrrFT1*. *BrrFT4* expression was also influenced by vernalization treatments, but the expression level was much lower than that of *BrrFT1* and *BrrFT2*. *BrrFT3* was still hardly detected before or after vernalization treatments. These results indicated that as *FT*-like genes, *BrrFT1* and *BrrFT2* were significantly induced by vernalization, and the *BFT*-like gene *BrrFT4* was also induced but to a lower degree.

### Promoter analysis of four *BrrFT* paralogues

**Fig. 4:**
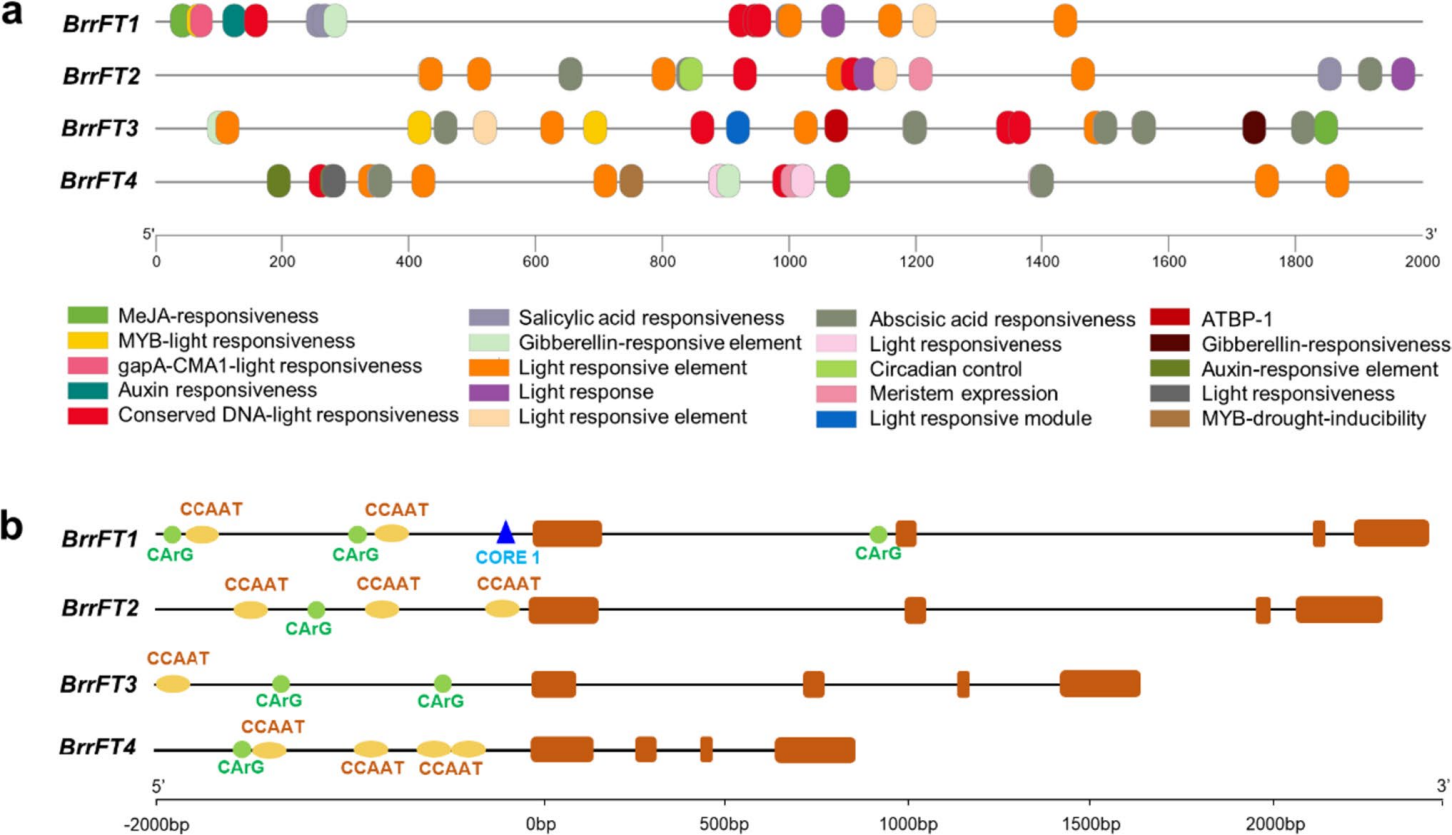
Promoter analysis of four *BrrFT* promoters. **a** Promoter cis-element analysis of four *BrrFT* promoters using the PlantCARE database web server. **b** The unique elements of the key regulators FLC, CO, and NF-Ys at the four *BrrFT* promoters. The 2 kb DNA fragments upstream of the starting code of *BrrFT* paralogues were analysed using the PlantCARE and PlantPAN web servers. CArG indicates the FLC binding site, CORE1 indicates the CO binding site, and CCAAT indicates the NF-Ys binding site

The expression levels of duplicated genes differ greatly due to genome duplication events and genome polyploidy, including expression level variations and spatiotemporal silencing (Flagel and Wendel 2009). To better understand the divergence in regulation of the four *BrrFT* paralogues, we further analysed the promoter sequences (2000 bp DNA sequence upstream of the ATG) among the four *BrrFT* paralogues. The cis-acting regulatory element prediction results showed that multiple cis-acting elements were found and were related to the light response and plant hormones, such as gibberellins (Gas), salicylic acid (SA), methyl jasmonate (MeJA), and auxin (Fig. 4a, Table S4), and the cis-regulatory elements among the four *BrrFT* promoters were significantly different. We further analysed the transcription factor binding sites (TFBS) of *BrrFT* promoter sequences. The results showed that almost 30 kinds of upstream TFs were predicted, including AP2, MADs box, NUCLEAR FACTOR-Ys (NF-Ys), and MYB family TFs (Table S5). We also analysed the unique elements of the key regulators FLC, CO, and NF-Ys at *BrrFT* promoter sequences (Fig. 4b). FLC binds to a CArG box in the promoter and first region of *FT* (Helliwell et al. 2006). We found that the CArG box was identified in the promoter of the four *BrrFT* sequences with different numbers among them. The CORE1 domain (Tiwari et al. 2010), in which CO binds to *FT*, was found in the promoter of *BrrFT1*. CCAAT elements via which NF-Ys bind to *FT* were also found in the four *BrrFT* promoters (Cao et al. 2014). The number and location of these regulating elements were different among the four *BrrFTs*, indicating that these four *BrrFT* paralogues were regulated by different upstream regulators.

We further analysed the expression levels of upstream (Fig. S3) of *BrrFT* genes before and after vernalization by RNA-sequencing analysis. The expression levels of key flower repressors of four *BrrFLC* paralogs greatly decreased after vernalization. We found that the expression levels of *BrrCO* were upregulated after vernalization, while the expression levels of most *BrrNF-Ys* paralogues were not significantly activated after vernalization. We also analysed the expression levels of downstream target genes (Fig. S4), great upregulation trends were observed in *AP1*, *SOC1*, and *LFY* paralogues by RNA-seq analysis. These results indicated that the differential binding sites and expression levels of the upstream regulatory elements of *FT* paralogues may be the reason for the differential expression levels of *FT* paralogues.

### Effect of *BrrFT* paralogues on flowering time in transgenic *Arabidopsis*

**Fig. 5:**
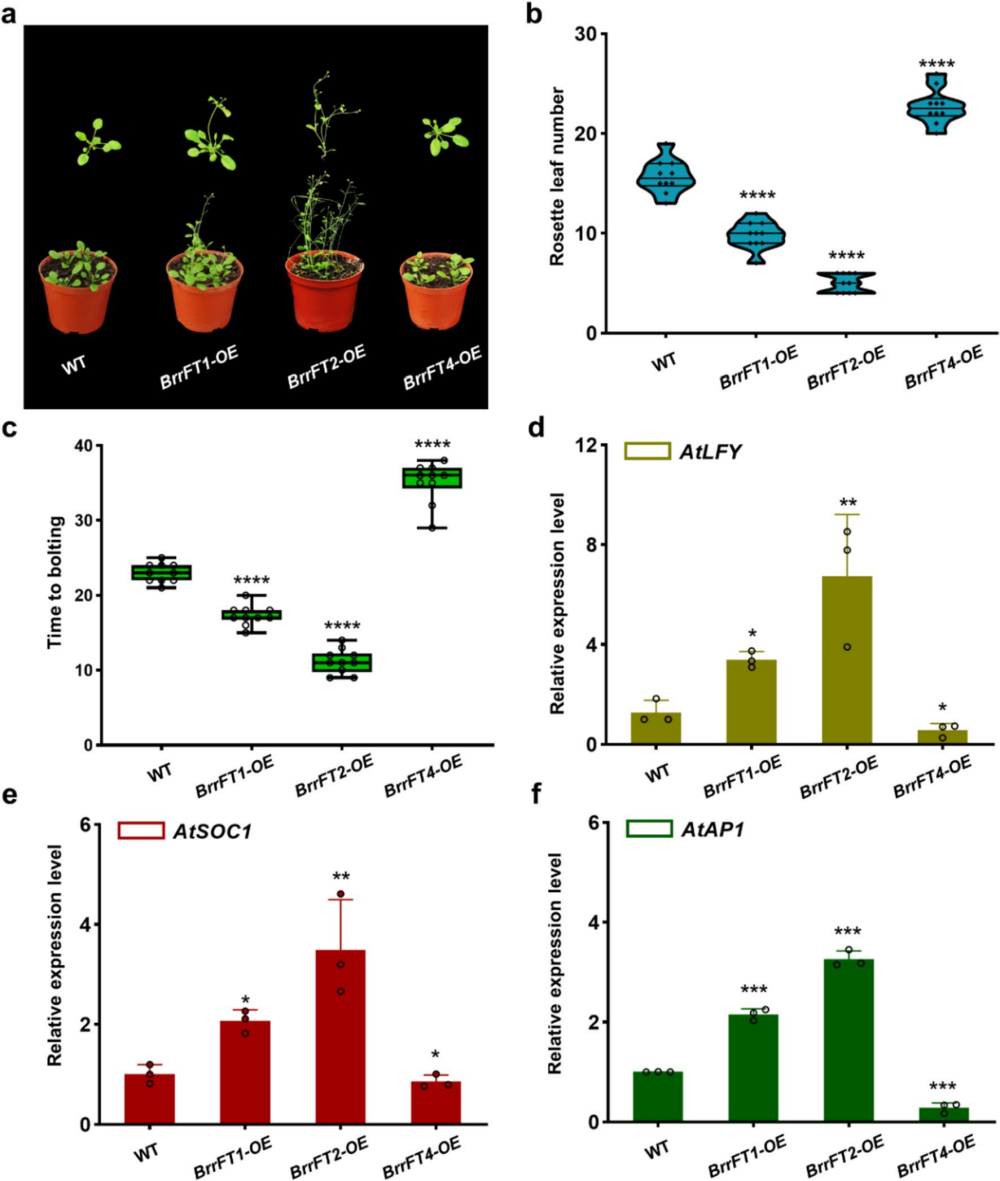
Overexpression of *BrrFT* homologues in *Arabidopsis*. **a** Representative phenotypes of transgenic plants overexpressing *BrrFT1*, *BrrFT2*, and *BrrFT4* paralogues. **b**–**c** Number of rosette leaves at flowering and number of days to flowering time of transgenic plants under long-day conditions. Data are mean ± SD, *n* = 10. **d**–**f** Expression levels of *AtLFY*, *AtSOC1*, and *AtAP1* in leaves of WT and transgenic *Arabidopsis*. The expression levels of *AtLFY*, *AtSOC1*, and *AtAP1* were normalized to that of *ACTIN2*. Data are mean ± SD, *n* = 3. Statistical analyses were performed using ordinary one-way ANOVA. ****, *P* < 0.0001; ***, *P* < 0.001; **, *P* < 0.01; *, *P* < 0.05

To further investigate the role of these *BrrFT* paralogues in regulating flowering time, we separately overexpressed the *BrrFT1*, *BrrFT2*, and *BrrFT4* genes in wild-type *Arabidopsis* and obtained the corresponding transgenic lines which were identified by western blotting (Fig. S5). We did not overexpress *BrrFT3*, due to the sequence mutations in *BrrFT3* and difficulty in detecting *BrrFT3* expression during all developmental stages in turnip, which indicated that *BrrFT3* may have lost its function in controlling flowering time. The transgenic plants carrying *BrrFT1* or *BrrFT2* flowered much earlier than the wild-type plants, and had fewer rosette leaves at bolting time (Fig. 5a–c, Fig. S6). Compared to wild-type *Arabidopsis*, which flowered with an average of 15.70 ± 1.70 total leaves after growing for 23.00 ± 1.25 days, *BrrFT2-OE* transgenic plants flowered significantly earlier with an average of 5.00 ± 0.94 total leaves after growing for 11.20 ± 1.61 days. In addition, *BrrFT1-OE* transgenic plants generated a moderate phenotype compared to *BrrFT2-OE* transgenic plants (with an average of 9.80 ± 1.40 total leaves after growing for 17.30 ± 1.34 days). However, the transgenic plants overexpressing *BrrFT4* showed a significant delay in flowering time (with an average of 22.70 ± 1.77 total leaves after growing for 35.10 ± 2.69 days) compared to wild-type plants. These results indicated that *BrrFT1* and *BrrFT2* maintained conserved functions in promoting flowering, and *BrrFT2* played a major role, with *BrrFT1* playing a lesser role in promoting flowering. However, *BrrFT4* possibly had an opposite effect in regulating flowering time.

Previous studies have reported that *LFY*, *SOC1*, and *AP1* are downstream genes regulated by FT, so the expression patterns of these FT downstream flowering-related genes were examined in transgenic plants (Goslin et al. 2017). As shown in Fig. 5d–f, the expression of *LFY*, *SOC1*, and *AP1* was significantly higher in *BrrFT2-OE* overexpressing plants than in wild-type plants, which was consistent with the earliest flowering phenotype. In addition, the expression levels of *LFY*, *SOC1*, and *AP1* in *BrrFT1*-overexpressing plants were moderately activated, higher than those in wild-type plants but lower than those in *BrrFT2-OE* plants. However, the expression levels of these genes in *BrrFT4-OE* transgenic plants were lower than those in wild-type plants (Fig. 5d–f, Fig. S7). These results indicated that *BrrFT1* and *BrrFT2* promoted floral transition by activating the expression of *LFY*, *SOC1*, and *AP1*, while *BrrFT4* inhibited flowering by repressing the expression of *LFY*, *SOC1*, and *AP1*.

### Genetic relationship of *BrrFT* paralogues mediating flowering time

**Fig. 6:**
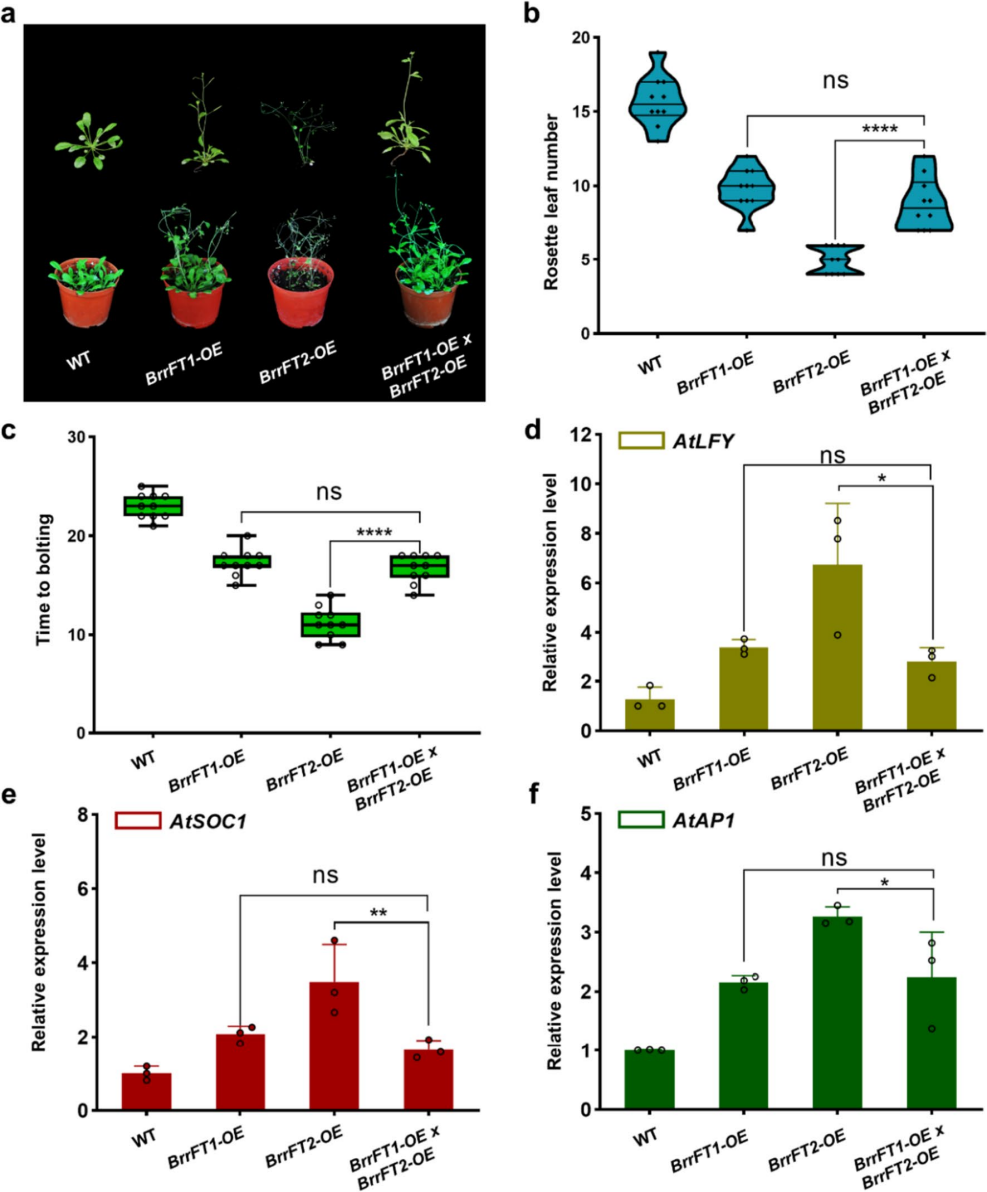
Analysis of the genetic relationships of *BrrFT1* and *BrrFT2* in flowering time regulation. **a** Phenotype analysis of F1 plants crossed with *BrrFT1-OE* and *BrrFT2-OE* transgenic lines. Data are mean ± SD, *n* = 10. **b**–**c** Number of rosette leaves at flowering and number of days to flowering time of F1 crossing plants under long-day conditions. **d**–**f** Expression patterns of *AtLFY*, *AtSOC1* and *AtAP1* in leaves of WT and F1 crossing plants. Data are mean ± SD, *n* = 3. Statistical analyses were performed using ordinary one-way ANOVA. ****, *P* < 0.0001; ***, *P* < 0.001; **, *P* < 0.01; *, *P* < 0.05

**Fig. 7:**
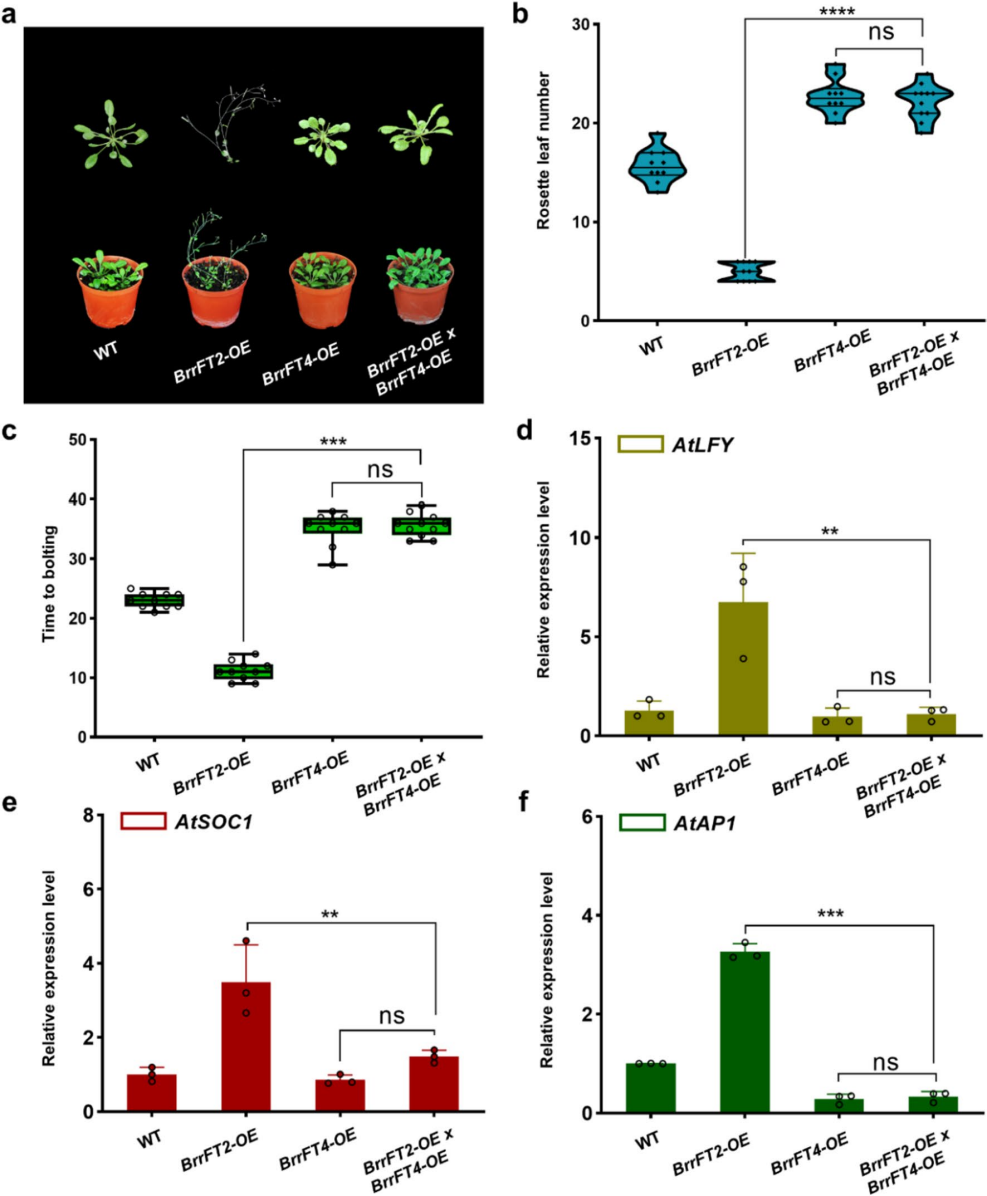
Analysis of the genetic relationships of *BrrFT2* and *BrrFT4* in flowering time regulation. **a** Phenotype analysis of F1 plants crossed with *BrrFT2-OE* and *BrrFT4-OE* transgenic lines. **b**–**c** Number of rosette leaves at flowering and number of days to flowering time of F1 crossing plants under long-day conditions. Data are mean ± SD, *n* = 10. **d**–**f** Expression patterns of *AtLFY*, *AtSOC1* and *AtAP1* in leaves of WT and F1 crossing plants. Data are mean ± SD, *n* = 3. Statistical analyses were performed using ordinary one-way ANOVA. ****, *P* < 0.0001; ***, *P* < 0.001; **, *P* < 0.01; *, *P* < 0.05

To investigate the genetic relationship among *BrrFT* homologous genes controlling flowering, we crossed *BrrFT2-OE* transgenic plants with *BrrFT1-OE* transgenic plants and *BrrFT4-OE* transgenic plants. The timing of flowering and the expression of downstream flowering genes were also measured in the F1 generation of transgenic *Arabidopsis*. We found that both *BrrFT1* and *BrrFT2* were highly expressed in F1 generation plants (Fig. S8), the flowering time of the F1 generation plants was as early as *BrrFT1-OE*, but not earlier as that of the *BrrFT2-OE* plants (Fig. 6a–c, Fig. S9), and the expression level of downstream genes was consistent with the flowering time (Fig. 6d–f, Fig. S10). These results indicated that there was no significantly additive effect between *BrrFT1* and *BrrFT2* in regulating flowering time in double-overexpression hybrid plants. *BrrFT2* and *BrrFT4* also highly expressed in F1 generation of *BrrFT2-OE/BrrFT4-OE* (Fig. S11), the F1 generation plants flowered with an average of 22.18 ± 1.78 leaves, similar to *BrrFT4-OE* plants. The flowering time of F1 plants was much later than that of the parental *BrrFT2-OE* plants (Fig. 7a–c, Fig. S12). The expression levels of the downstream genes *LFY*, *SOC1*, and *AP1* were similar to those of *BrrFT4-OE* plants (Fig. 7d–f, Fig. S13), indicating an epistatic effect of *BrrFT4* on *BrrFT2* in double-overexpression crossing lines in *Arabidopsis*. Together, these findings suggested that *BrrFT1*, *BrrFT2*, and *BrrFT4* collectively played roles in regulating flowering time.

### BrrFT4 delays flowering by competing with BrrFT2 and BrrFT1 for binding BrrFD proteins

**Fig. 8:**
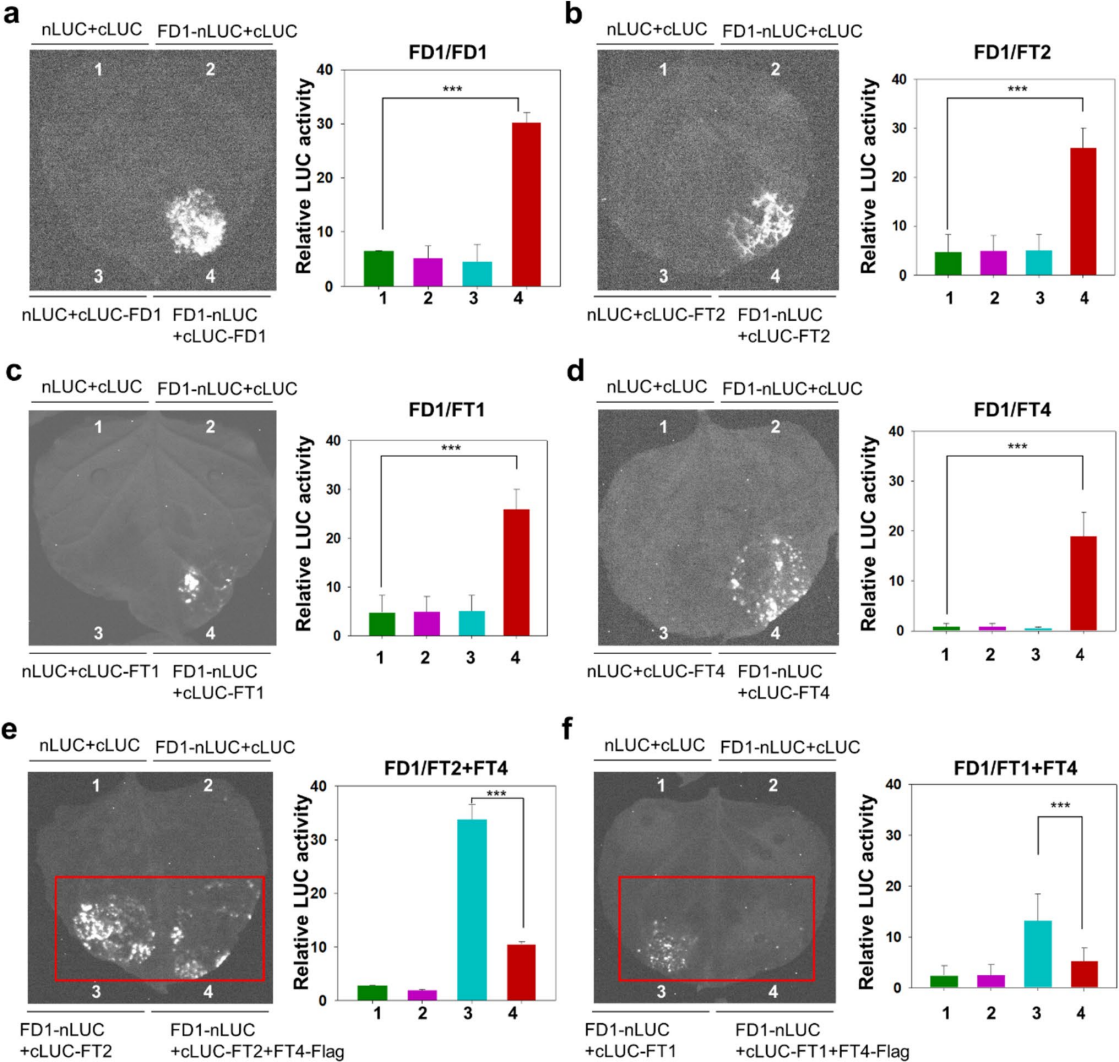
LCI assays between BrrFT1, 2, 4 and BrrFD1 in *N. benthamiana* leaves. **a** LCI assays between BrrFD1 and BrrFD1 in *N. benthamiana* leaves. The interaction signal was observed between BrrFD1-nLUC and cLUC-BrrFD1. **b** LCI assays between BrrFT2 and BrrFD1. **c** LCI assays between BrrFT1 and BrrFD1. **d** LCI assays between BrrFT4 and BrrFD1. **e** LCI assays to identify the relationship of BrrFT2 and BrrFT4 on interacting with BrrFD1. **f** LCI assays to identify the relationship of BrrFT1 and BrrFT4 interacting with BrrFD1. Relative LUC activity is displayed next to the image. Data are the mean ± SD. *n* = 5. Statistical analyses were performed using ordinary one-way ANOVA. ***, *P* < 0.001

It was reported that BFT inhibits flowering by competing with FT for interacting with FD in the floral transition in *Arabidopsis* (Ryu et al. 2014). In this study, we found that the expression of the *BFT*-like paralogue *BrrFT4* was induced by vernalization and floral transition, although it was significantly lower than that of *BrrFT2* in turnip. We speculated that the flowering-delaying activity of BrrFT4 may compete with the promotive activities of BrrFT1 and BrrFT2 via competitive interaction with FD. To verify this hypothesis, three *BrrFD* homeologs were first identified in turnip based on the *FD* homeologs in the BRAD. We then cloned the most conserved *BrrFD* paralogues compared to the *Arabidopsis* FD gene, termed *BrrFD1* and *BrrFD2* (Fig. S14, Table S6). We then inserted *BrrFD1* and *BrrFD2* into nLUC and cLUC vectors, respectively. Equal volumes of *A. tumefaciens* containing BrrFD1-nLUC and BrrFD1-cLUC, BrrFD2-nLUC and BrrFD2-cLUC constructs were mixed and co-injected into *N. benthamiana* leaf epidermal cells for luciferase complementation imaging (LCI) assays. The results showed that BrrFD1 and BrrFD2 can actually interact on their own (Fig. 8a and S15a). Furthermore, we inserted *BrrFT1*, *BrrFT2*, and *BrrFT4* into the cLUC vector, respectively. Strong interaction signals were detected when BrrFT2-cLUC and BrrFD1-nLUC or BrrFT2-cLUC and BrrFD2-nLUC were fused and transiently coexpressed in *N. benthamiana* leaves (Fig. 8b and S15b). In addition, similar results were also detected between BrrFT1-cLUC and BrrFD1-nLUC, BrrFT4-cLUC and BrrFD1-nLUC, BrrFT1-cLUC and BrrFD2-nLUC, and BrrFT4-cLUC and BrrFD2-nLUC with different relative LUC activities (Figs. 8c–d and S15c-d), compared with the injection of empty or single construct combinations that were not detected the fluorescence. These results suggested that the three BrrFT proteins were able to interact with BrrFD1 and BrrFD2 in a similar manner.

Furthermore, to explore whether BrrFT4 can competitively interact with BrrFD homeologs, equal volume of *A. tumefaciens* containing the *35S:BrrFT4-Flag* plasmid was added into the BrrFT2-cLUC and BrrFD1-nLUC, BrrFT1-cLUC and BrrFD1-nLUC, BrrFT2-cLUC and BrrFD2-nLUC or BrrFT1-cLUC and BrrFD2-nLUC injection. We used the empty vectors of pCAMBIA1300-cLUC and pCAMBIA1300-nLUC as negative control, which were not detected the LUC signal after injection. Our results showed that weakened fluorescence was detected in the combination of BrrFT2-cLUC and BrrFD1-nLUC (or BrrFD2-nLUC) with BrrFT4-Flag, compared to that without BrrFT4-Flag (Fig. 8e and S15e). Similar weaken signal was also detected when co-injected BrrFT1-cLUC and BrrFD1-nLUC (or BrrFD2-nLUC) with BrrFT4-Flag, compared to that without BrrFT4-Flag (Fig. 8f and S15f). The results clearly showed that BrrFT4 can block BrrFT2 and BrrFT1 from interacting with BrrFD1 and BrrFD2. These findings demonstrated that as a BFT-like protein, BrrFT4 can compete with BrrFT2 and BrrFT1 for competitive binding to BrrFD proteins, and finally antagonistically affect BrrFT2 and BrrFT1 in flowering promotion.

## Discussion

After divergence from *Arabidopsis*, ancestral species of *Brassica* evolved through WGT events that resulted in subgenome divergence and the possible existence of multiple homologous genes of *Brassica*, which made the gene regulatory networks more complex (Cheng et al. 2014, 2018). Instead of extended gene copy number, homologues retained conserved functions or evolved sub- and neo-functionalization via different temporal expression patterns and facilitated plant adaptive evolution (Roulin et al. 2013). FT functions in the floral transition process as a transcriptional activator, and previous studies have proven that the role of the FT gene in flowering time regulation seems to be conserved among different species (Wickland and Hanzawa 2015; Jin et al. 2021). Due to the evolutionary history of the *Brassica* genus, several copies of floral integrator homologues exist in the *Brassica* genus, so the function of *FT* paralogues is more complex. It has been reported that five *FT-*related *BnaTFL1* paralogues were isolated in *Brassica napus* and displayed different expression patterns. *BnaC03. TFL1 is* involved in negatively mediating flowering time, and all five *BnaTFL1* paralogues participate in plant morphogenesis (Sriboon et al. 2020). In this study, 12 *FT*-related paralogues were identified in turnip, and four of these genes which shared high identities with *BraFT* genes were investigated for functional differences in flowering time. Genetic and biochemical experiments demonstrated that these genes cooperatively functioned in flowering time regulation in plant.

Previous studies showed that the divergence and duplication of *FT*-like genes were affected by domestication in cereals, and *FT*-related genes were targets for artificial selection (Qin et al. 2019). *Brassica rapa* ssp. *rapa* is a winter-annual *Brassica* plant that exhibits a vernalization requirement for flowering. FT was supposed to act as a floral integrator connecting the vernalization and photoperiod pathways (Takagi et al. 2023). We found that the expression level of *BrrFT* paralogues in the vegetative period without vernalization was low, while the expression was greatly induced after vernalization, except *BrrFT3*. Reportedly, the expression differences of duplicated genes are related to functional diversification (Pin and Nilsson 2012). In this study, our results showed that four *BrrFT* paralogues exhibited different expression levels and patterns. The high expression levels of *BrrFT1* and *BrrFT2* may contribute to facilitate the floral transition of turnip. Transcription factors play important roles in plant developmental progress by regulating downstream target genes binding to cis-elements in the promoter of specific target genes. FT is a floral integrator that various transcription factors induce or repress its expression level via binding to certain cis-elements distributed on its promoter. In this study, we identified binding motifs of NF-Ys, MYB, and MAD-box TF families and the specific elements of FLC, CO, and NF-Ys distributed on the promoters of *BrrFTs*. These results suggested that the *BrrFT* paralogues may play different roles in regulating flowering time via different expression levels in turnip.

*FT*-related genes have been reported to evolve into floral promoters and repressors during the functional diversification process (Pin and Nilsson 2012; Jin et al. 2021). Our genetic experiments revealed that *BrrFT2* overexpressing plants showed a strong early flowering phenotype, consistent with the highest expression level in floral initiation stage of turnip, suggesting that BrrFT2 acted as the key floral inducer in turnip. In addition. BrrFT1 seems to act as a mild ‘florigen’ protein, with a mild early flowering phenotype in *BrrFT1*-overexpressing transgenic plants. In contrast, overexpressing *BrrFT4* in *Arabidopsis* resulted in significantly late flowering, indicating that it acted as a transcriptional repressor of flowering. Several conserved amino acids are found to distinguish inducer or repressor functions in FT-related proteins; most of the inducer FT homoeologs contained tyrosine (Y) at position 134 and tryptophan (W) at position 138, and all repressor FT homoeologs contained nontyrosine amino acids at position 134 and nontryptophan at position 138 (Wickland and Hanzawa 2015; Jin et al. 2021). Sequence blast analysis revealed that BrrFT1 and BrrFT2 paralogues retained conserved amino acids at positions 134Y and 138W, while in BrrFT4, these residues changed to 134N and 138Q, suggesting that the functions of the BrrFT paralogues diverged.

FT-like paralogues are crucial for plant development, especially flowering time regulation in different species. A previous study found that GmFT5a, GmFT2a, GmFT3b, and GmFT5b functioned as floral initiators in soybean, and GmFT5a exhibited a decisive effect in promoting flowering time (Liu et al. 2018). *FT* genes have evolved multiple functions due to duplication and diversified functional changes during the evolution of various crops. FT genes have gradually evolved to repress flowering, and some FT paralogues act antagonistically in some crops, such as sugar beet, onion, and tobacco (Lee et al. 2013; Jin et al. 2021). Our results showed that *BrrFT4* had an epistatic effect on *BrrFT2*, resulting in late flowering in transgenic plants with double-overexpression of *BrrFT4* and *BrrFT2*. In addition, the expression levels of both *BrrFT2* and *BrrFT4* were induced by vernalization and floral transition in turnip, but the expression level of *BrrFT4* was much lower than that of *BrrFT2*. Thus, we proposed that as a floral repressor, *BrrFT4* plays a role in antagonistically regulating flowering time via transcript level variation. These data showed that four *BrrFT* paralogues cooperatively functioned in regulation flowering time of turnip. It has shown that BFT delayed flowering time by competitively binding to FD with FT (Ryu et al. 2014). Our molecular biochemical experiments demonstrated that BrrFT4 can weaken the interaction capacities of BrrFT1 and BrrFT2 to bind to BrrFD proteins via competitively interacting with BrrFD proteins, which further demonstrated that BrrFT4 played an important role in repressing flowering time.

Together, our results revealed the functional divergence of *BrrFT* paralogues in flowering time regulation in turnip. Our findings provide insight into the role of *BrrFT* paralogues in fine-tuning the flowering time in turnip in response to environmental changes after WGT events and artificial domestication.

### Supplementary Information

Below is the link to the electronic supplementary material.

Supplementary file1 (DOCX 7622 KB)

Supplementary file2 (XLSX 11 KB)

Supplementary file3 (XLSX 9 KB)

Supplementary file4 (XLSX 10 KB)

Supplementary file5 (XLSX 12 KB)

Supplementary file6 (XLSX 10 KB)

Supplementary file7 (XLSX 9 KB)

## Data Availability

Reference transcriptome data are deposited in the SCIENCE DATA BANK with a private link view: https://www.scidb.cn/s/FR7NRz.
