# Supplementary material for: The evolution and functional divergence of FT-related genes in controlling flowering time in Brassica rapa ssp. rapa
Source: Plant Cell Rep. 2024 Mar 7;43(4):86. doi: 10.1007/s00299-024-03166-2 (PMC10920429; doi:10.1007/s00299-024-03166-2)
Supplement: Supplementary file 1 — Supplementary file1 (DOCX 7622 KB) [file 299_2024_3166_MOESM1_ESM.docx]

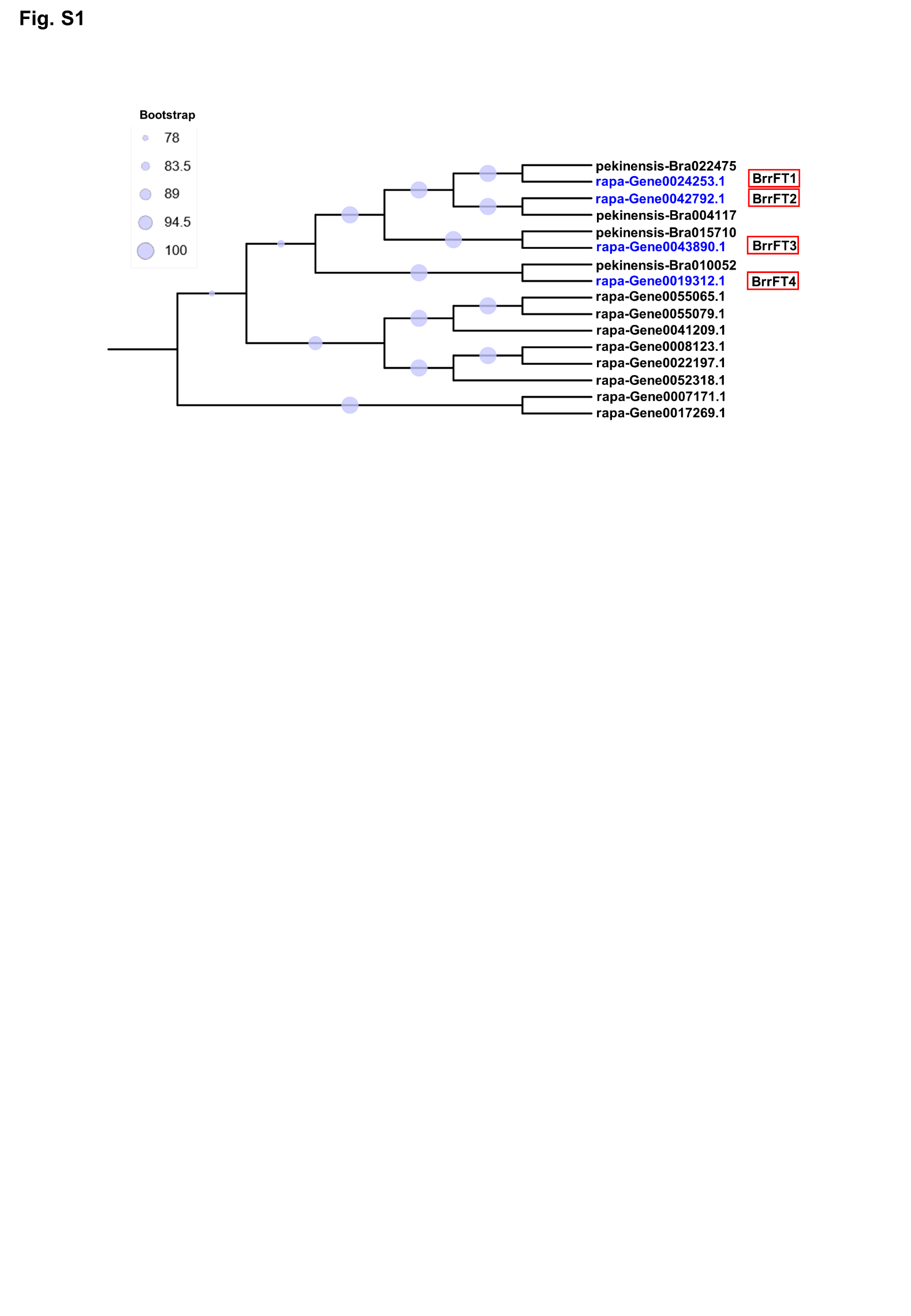


**Fig. S1 Phylogenetic tree of four BraFT proteins in BRAD and FT-related proteins in turnip.** The tree was constructed based on full-length protein sequences of *FT*-related genes using the neighbour joining method in TreeBeST v.0.2.0. Each name contains the name of the plant variety and the gene accession number in the Genome Warehouse database. Specifically, pekinensis, *B. rapa* ssp. *pekinensis*; rapa, *B.rapa* ssp. *rapa*.


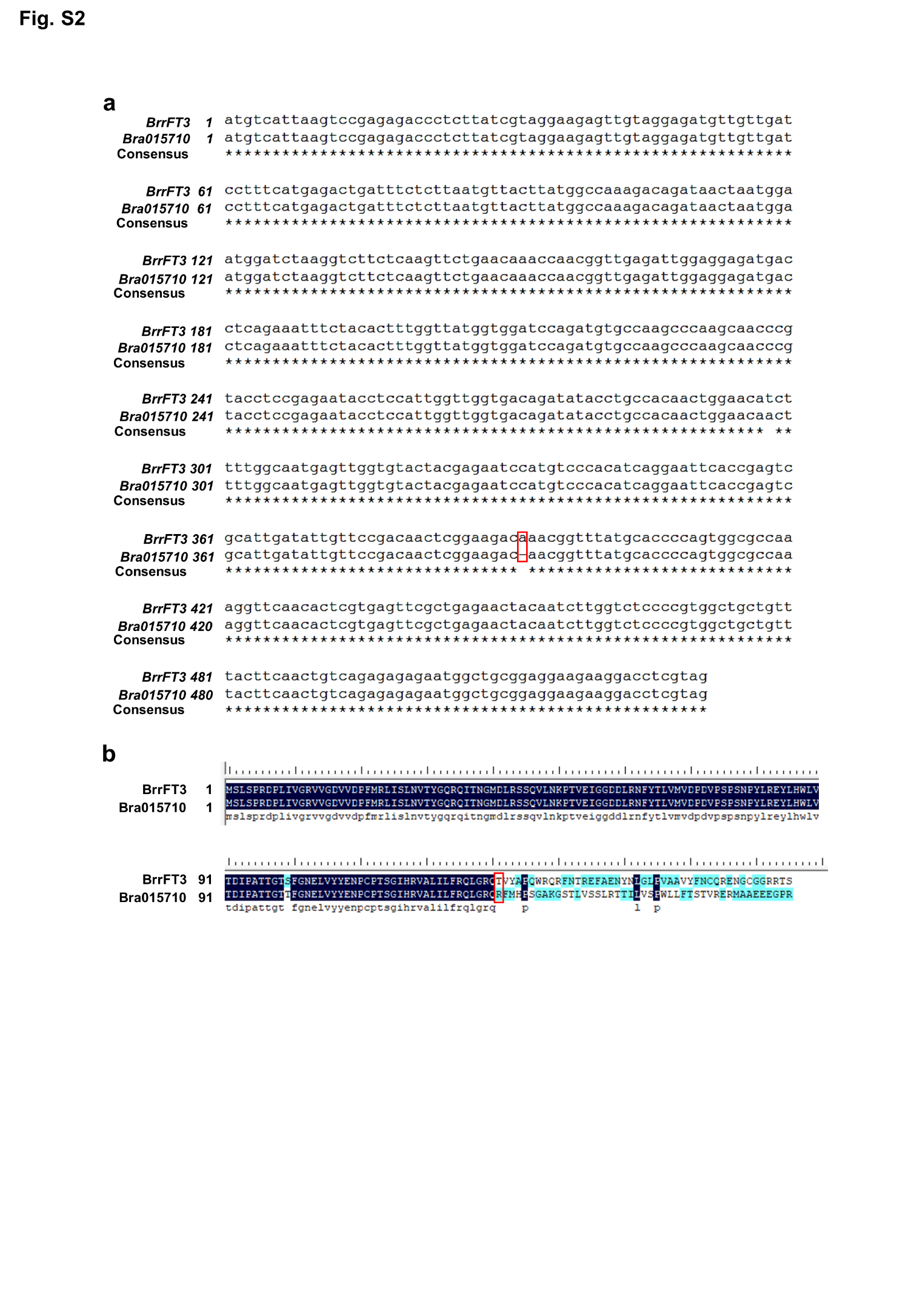


**Fig. S2. Alignment analysis of the *BrrFT3* gene with its native homologue in Chinese cabbage (*Bra015710*).** (a) Gene sequence alignments between *BrrFT3* and *Bra015710*. (b) Protein sequence alignments between BrrFT3 and Bra015710.


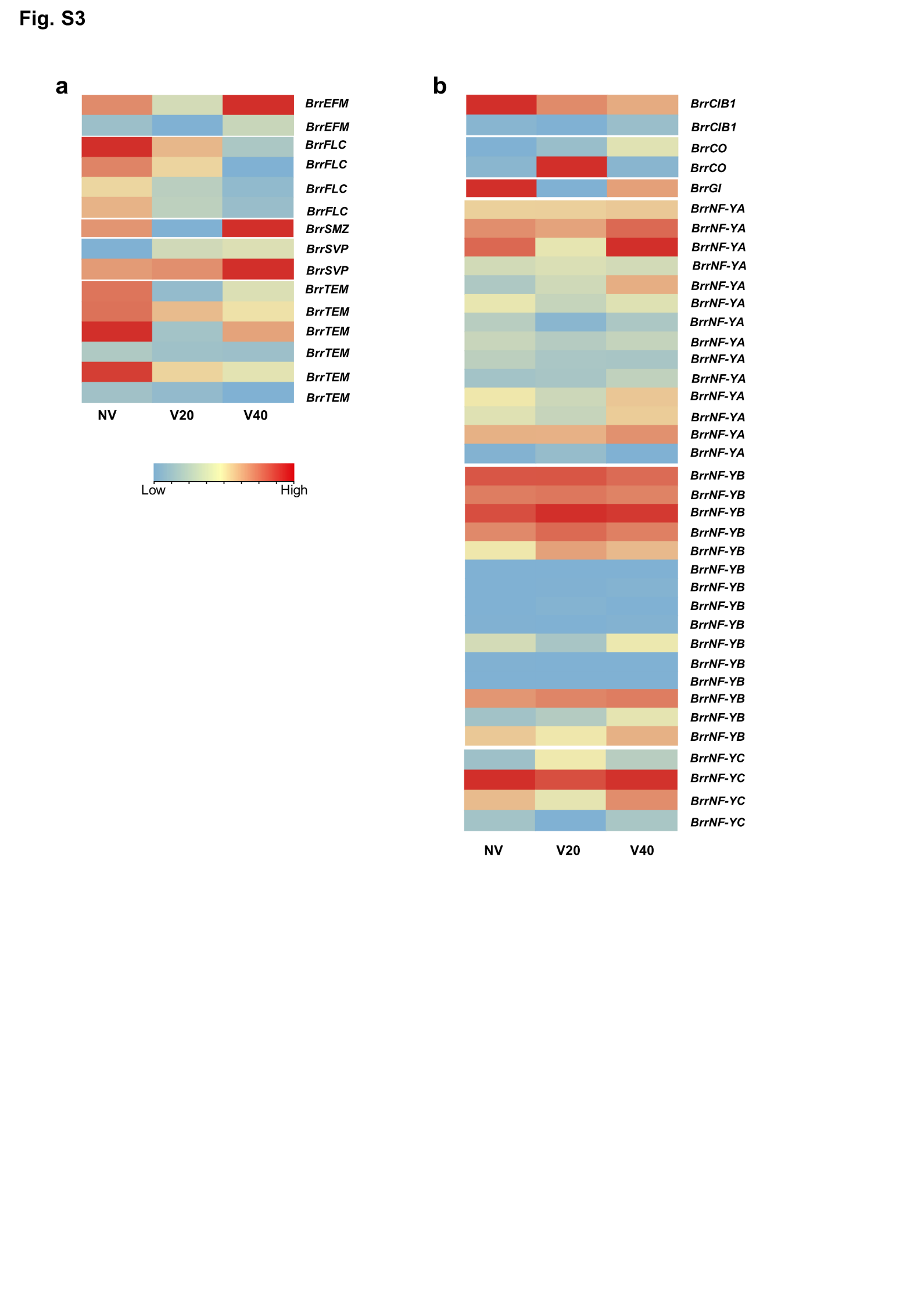


**Fig. S3. Heatmap analysis of FT-upstream targeted genes in turnip before and after vernalization.** (a) Heatmap showing the expression patterns of activators upstream four *BrrFT* paralogues before and after vernalization based on the RNA-seq. (b) Heatmap showing the expression patterns of repressors upstream four *BrrFT* paralogues before and after vernalization. The scale bar indicates the log2 transformed FPKM values.


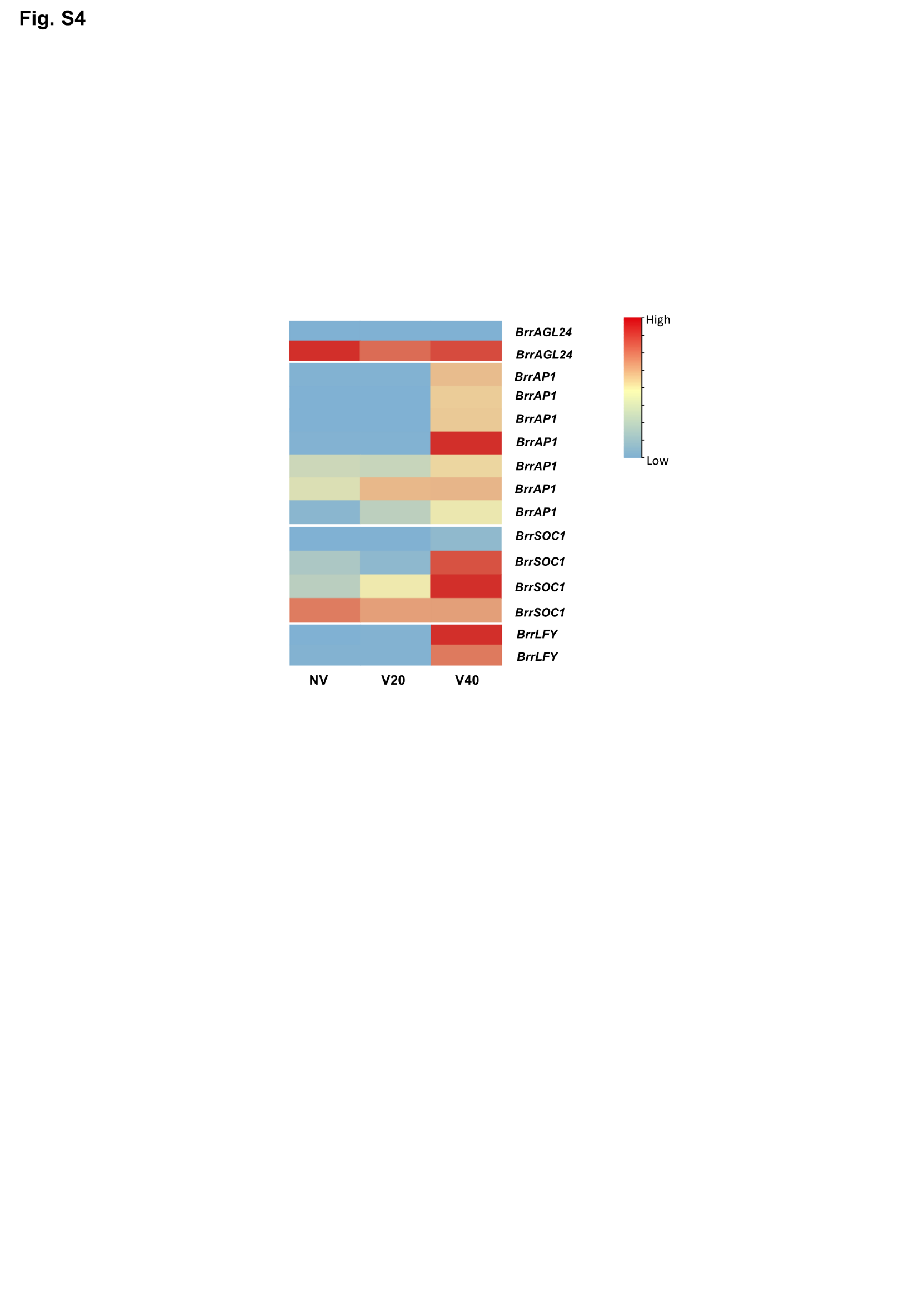


**Fig. S4. Heatmap analysis of FT-downstream targeted genes in turnip** **before and after vernalization.** Forty-day vernalization treatment is sufficient to complete flower transition. The heatmap was based on the RNA-seq transcriptome database in *B. rapa* ssp*. rapa*. The scale bar indicates the log2 transformed FPKM values.


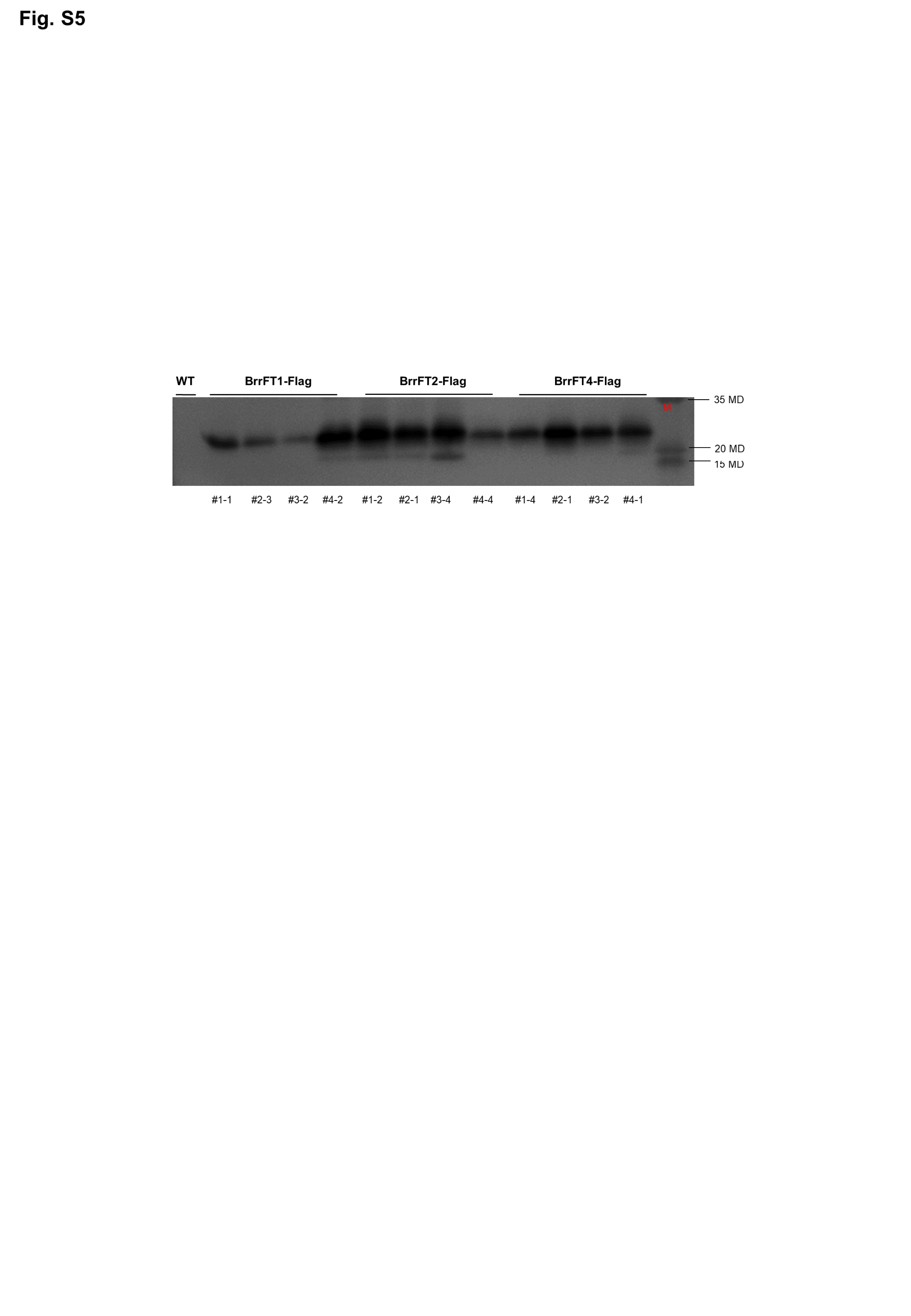


**Fig. S5. Validation of the *BrrFT* overexpression plants by western blot analysis.** Anti-Flag antibody was used to immunoprecipitate the proteins.


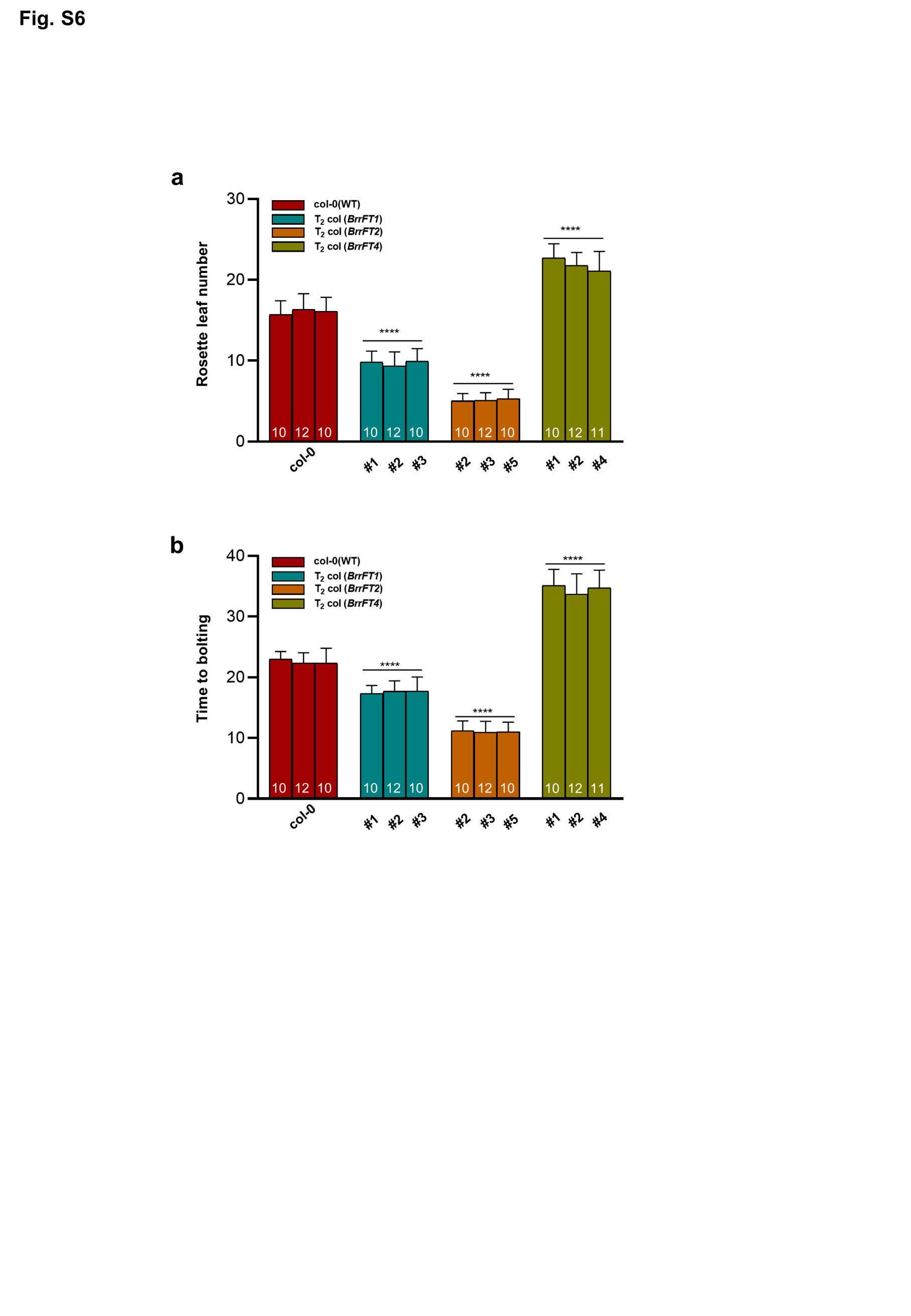


**Fig. S6.** Flowering time analysis of transgenic plants under long-day conditions. (a) Number of rosette leaves at flowering time of transgenic plants. (b) Number of days to flowering time of transgenic plants. Data are mean ±SD, n≥10.The number in the bar indicates the number of plants used of analysis. Statistical analyses were performed using ordinary one-way ANOVA. ****, P<0.0001.


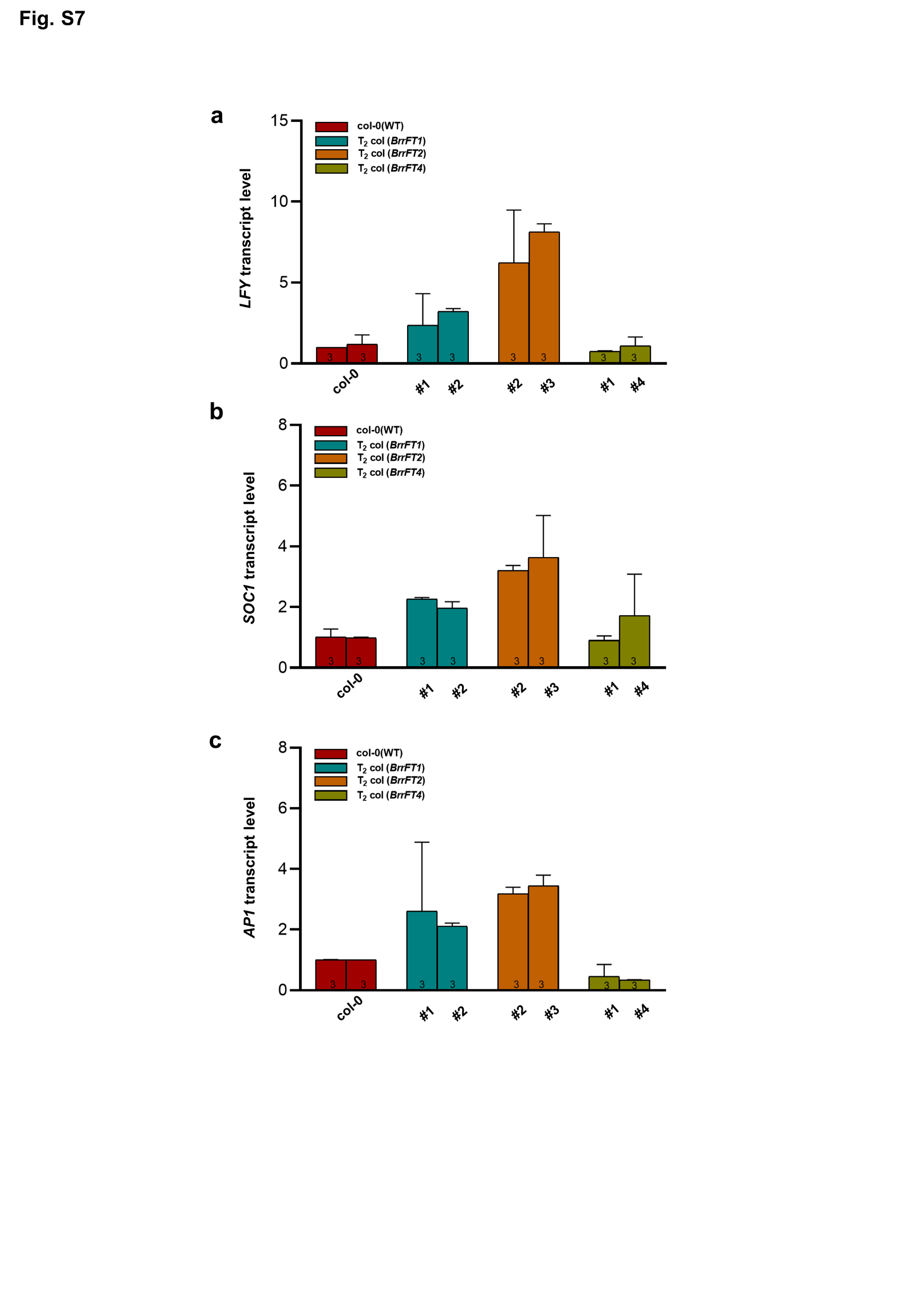


**Fig. S7. Expression levels of *AtLFY*, *AtSOC1* and *AtAP1* in leaves of WT and transgenic *Arabidopsis*.** (a) Expression levels of *AtLFY.* (b) Expression levels of *AtSOC1.* (c) Expression levels of AtAP1. The expression levels of *AtLFY*, *AtSOC1* and *AtAP1* were normalized to that of *ACTIN2*. Data are mean ±SD, n=3.


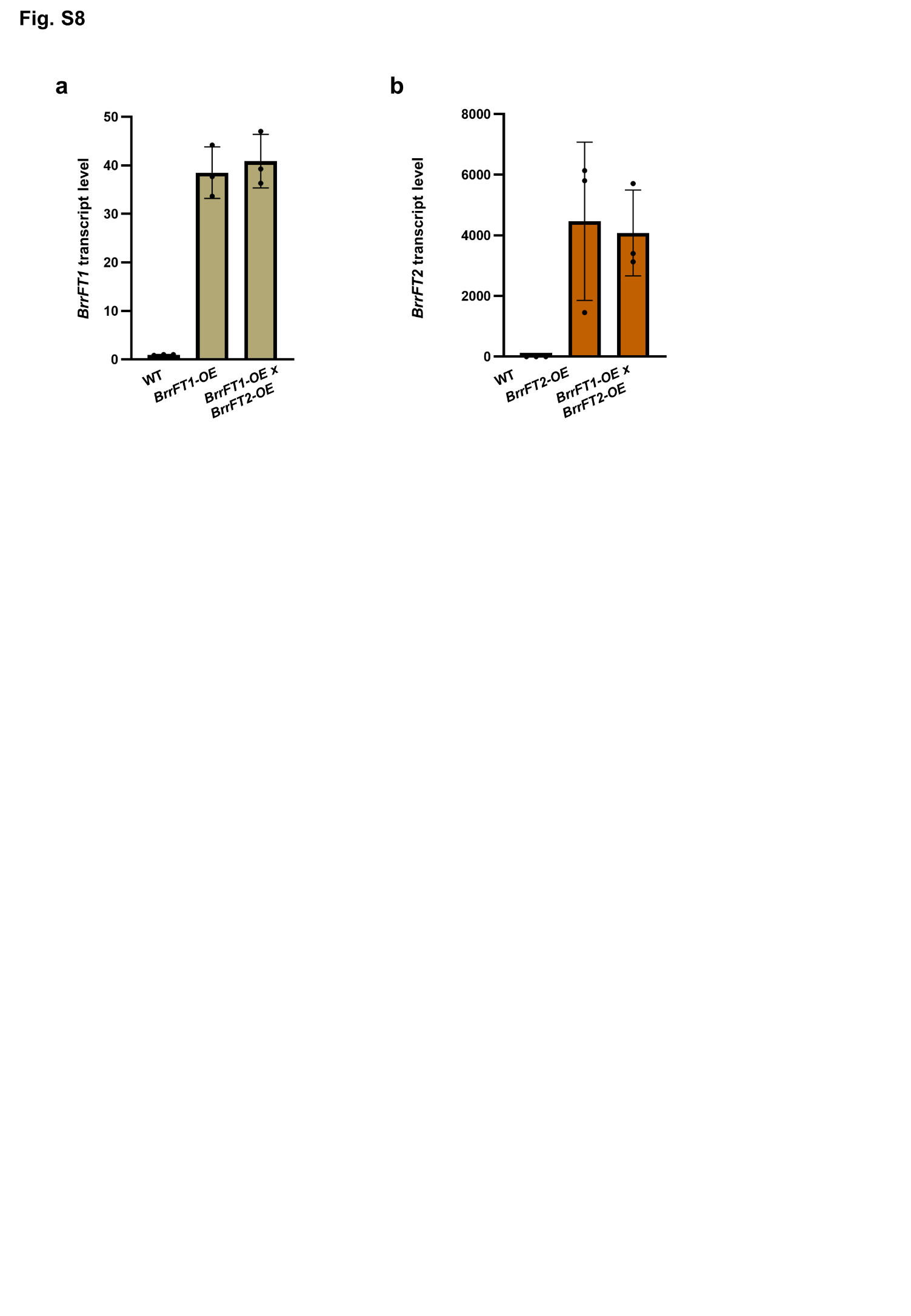


**Fig. S8. Validation of the F1 plants crossed by *BrrFT1-OE* and *BrrFT2-OE* transgenic lines by** **qRT‒PCR.** (a) The expression level of *BrrFT1* in parent *BrrFT1-OE* and F1 crossing plants. (b) The expression level of *BrrFT2* in parent *BrrFT2-OE* and F1 crossing plants. The expression levels of *BrrFT* paralogues were normalized to that of *ACTIN2*. Data are mean ±SD, n=3.


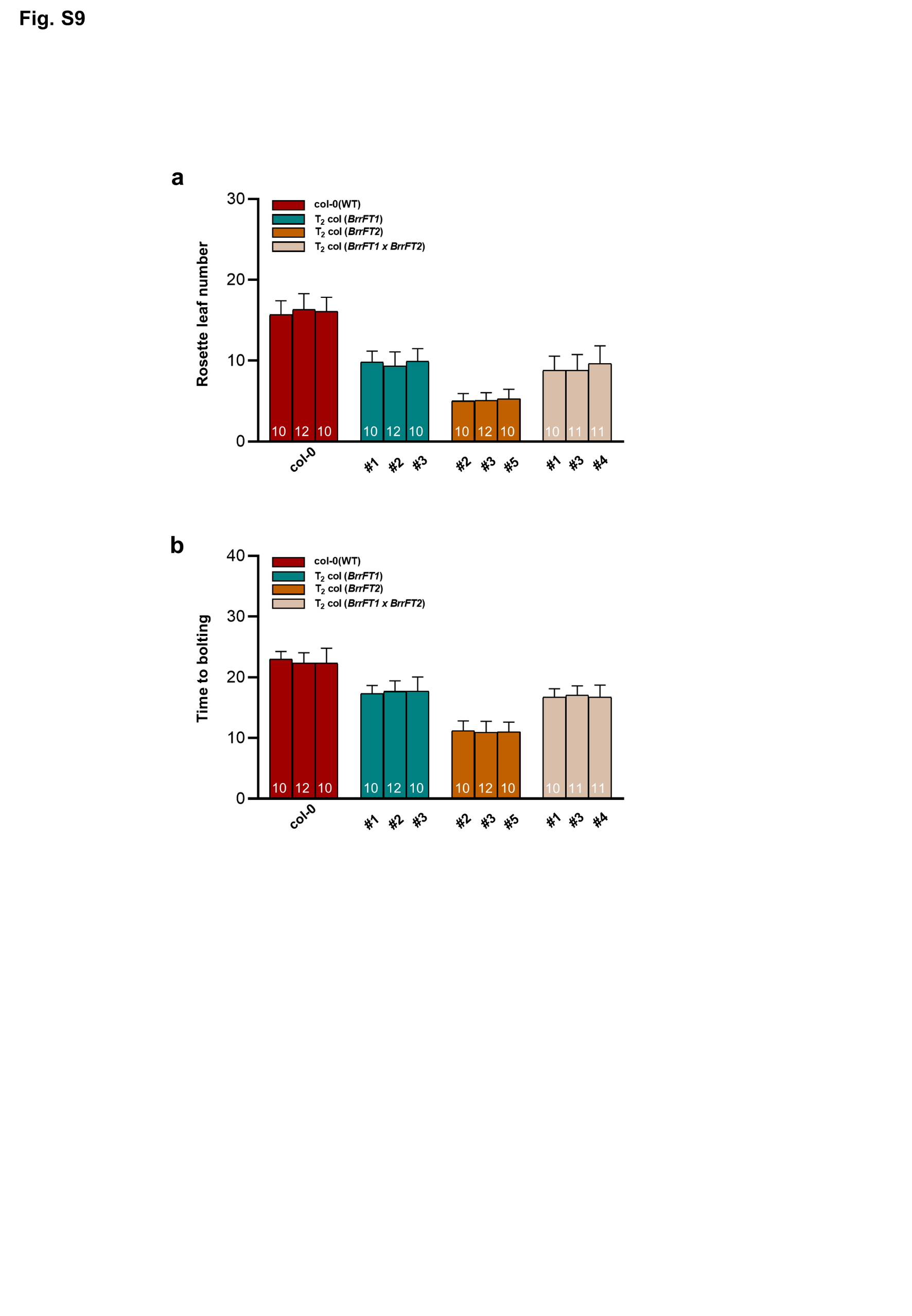


**Fig. S9. Flowering time analysis of F1 plants crossed with *BrrFT1-OE* and *BrrFT2-OE* transgenic lines under long-day conditions.** (a) Number of rosette leaves at flowering time of F1 plants crossed with *BrrFT1-OE* and *BrrFT2-OE* transgenic plants. (b) Number of days to flowering time of F1 plants crossed with *BrrFT1-OE* and *BrrFT2-OE* transgenic plants. Data are mean ±SD, n≥10. The number in the bar indicates the number of plants used of analysis.


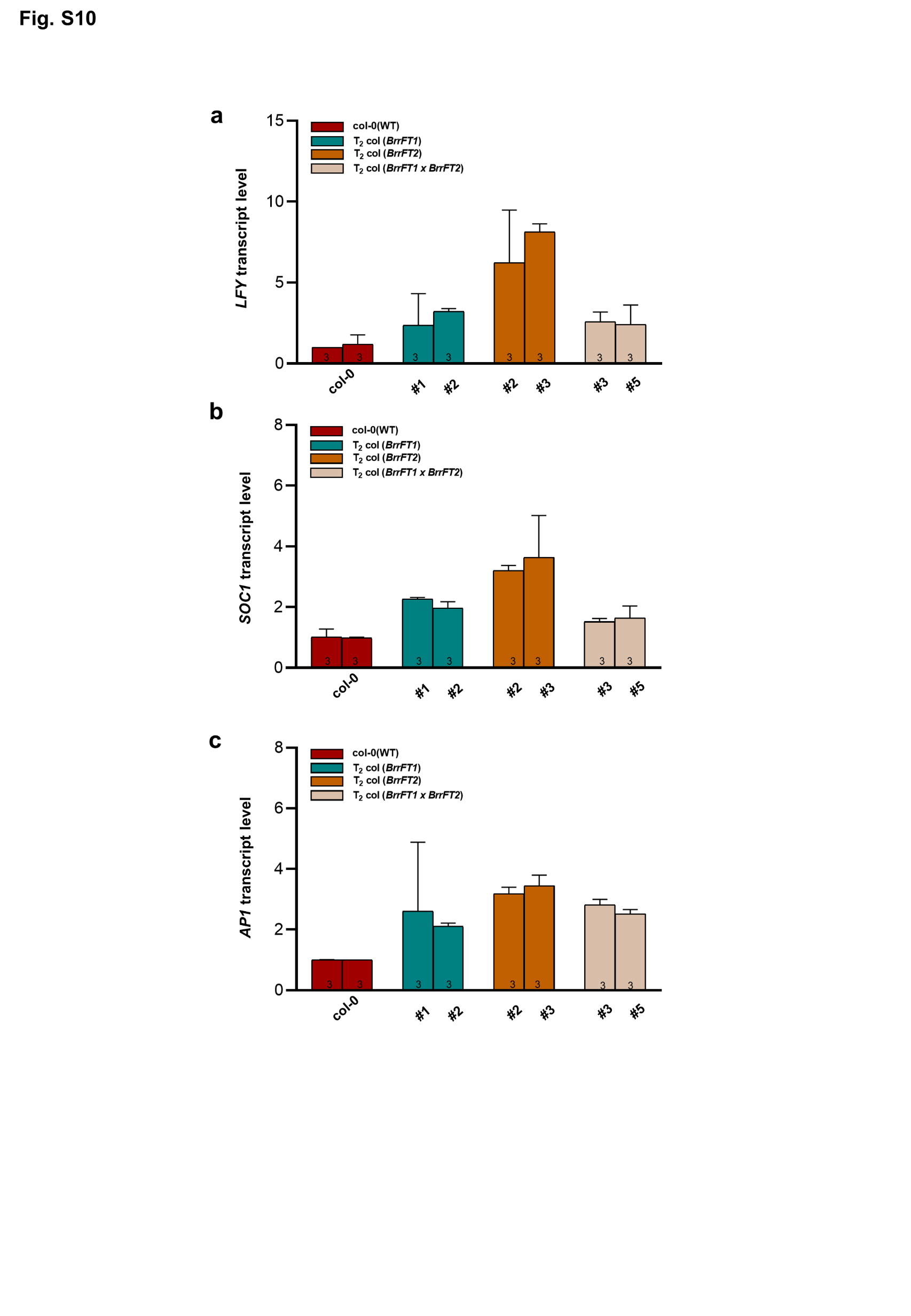


**Fig. S10. Expression levels of *AtLFY*, *AtSOC1* and *AtAP1* in leaves of WT and F1 plants crossed with *BrrFT1-OE* and *BrrFT2-OE* transgenic lines.** (a) Expression levels of *AtLFY.* (b) Expression levels of *AtSOC1.* (c) Expression levels of AtAP1. The expression levels of *AtLFY*, *AtSOC1* and *AtAP1* were normalized to that of *ACTIN2*. Data are mean ±SD, n=3.


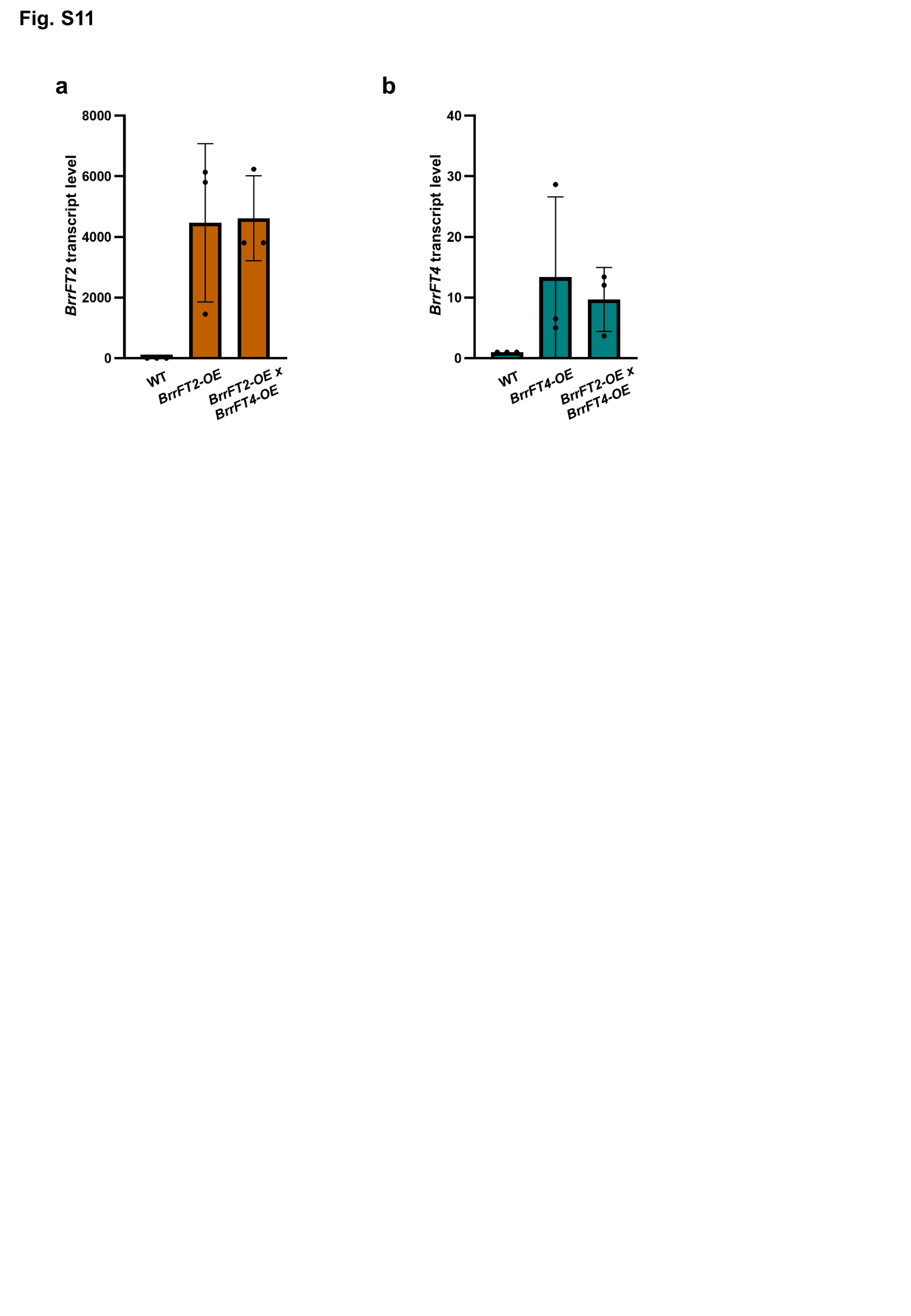


**Fig. S11. Validation of the F1 plants crossed by *BrrFT2-OE* and *BrrFT4-OE* transgenic lines by qRT‒PCR.** (a) The expression level of *BrrFT2* in parent *BrrFT2-OE* and F1 crossing plants. (b) The expression level of *BrrFT4* in parent *BrrFT4-OE* and F1 crossing plants. The expression levels of *BrrFT* paralogues were normalized to that of *ACTIN2*. Data are mean ±SD, n=3.


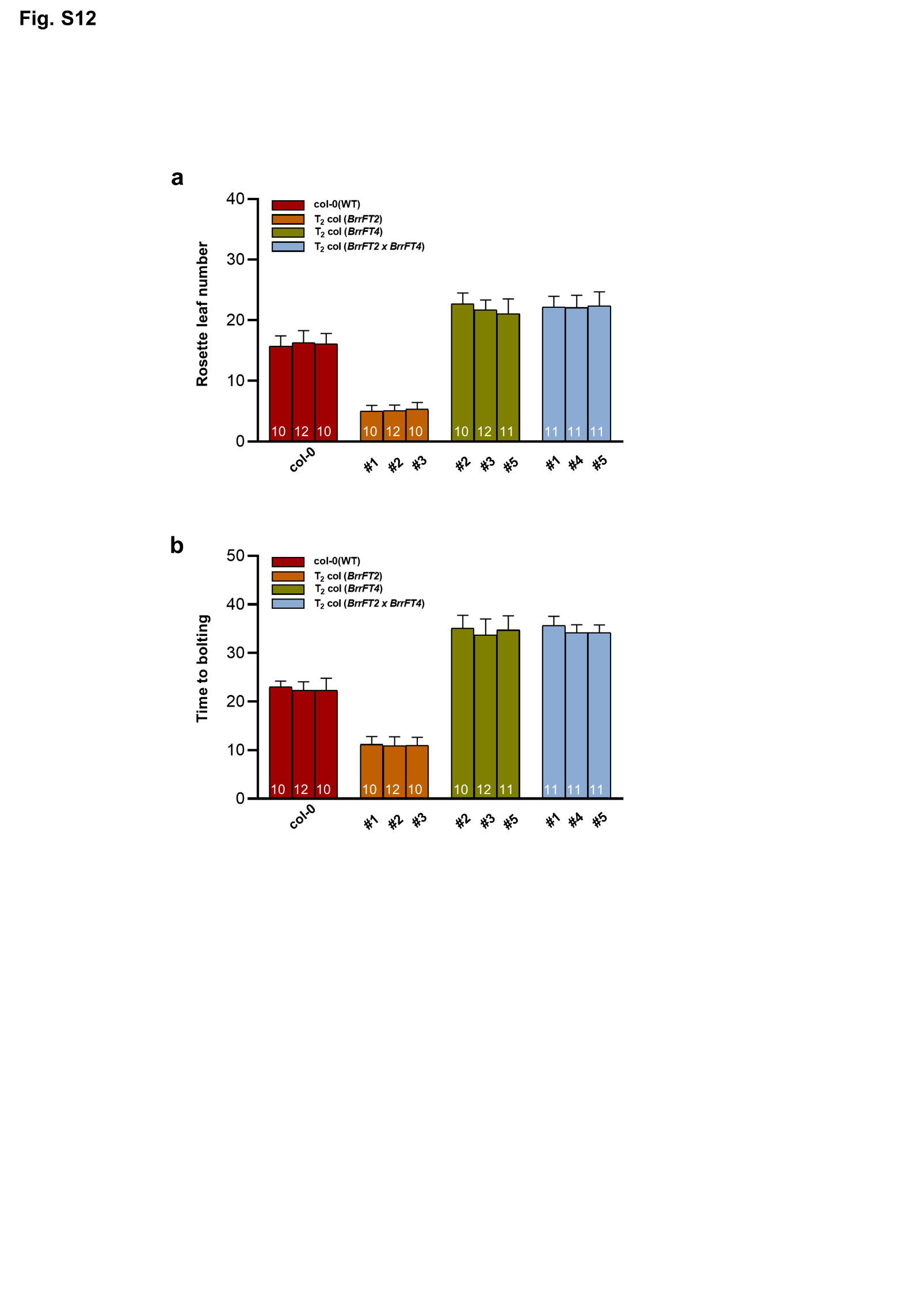


**Fig. S12. Flowering time analysis of F1 plants crossed with *BrrFT2-OE* and *BrrFT4-OE* transgenic lines under long-day conditions.** (a) Number of rosette leaves at flowering time of F1 plants crossed with *BrrFT2-OE* and *BrrFT4-OE* transgenic plants. (b) Number of days to flowering time of F1 plants crossed with *BrrFT2-OE* and *BrrFT4-OE* transgenic plants. Data are mean ±SD, n≥10. The number in the bar indicates the number of plants used of analysis.


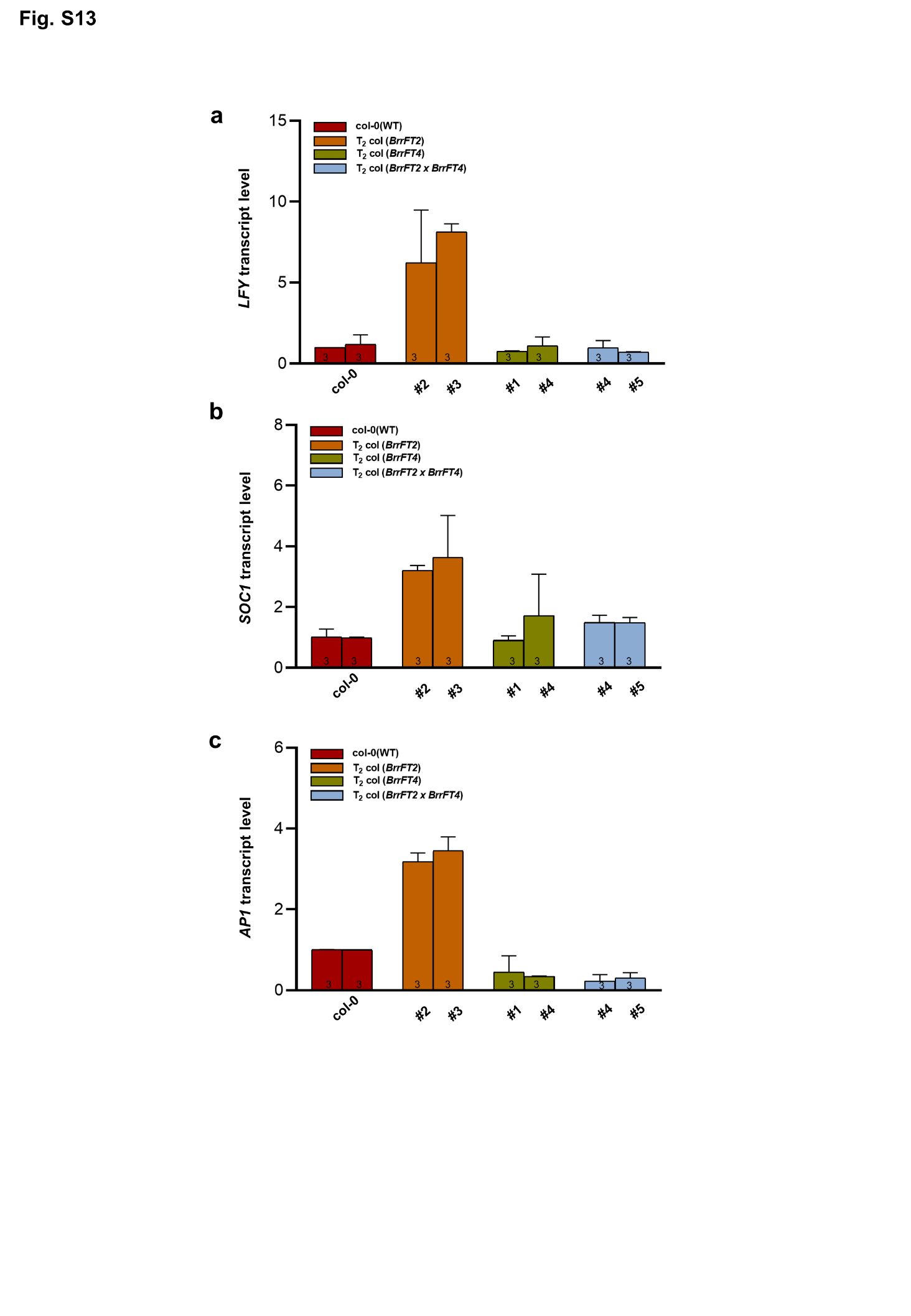


**Fig. S13. Expression levels of *AtLFY*, *AtSOC1* and *AtAP1* in leaves of WT and F1 plants crossed with *BrrFT2-OE* and *BrrFT4-OE* transgenic lines.** (a) Expression levels of *AtLFY.* (b) Expression levels of *AtSOC1.* (c) Expression levels of AtAP1. The expression levels of *AtLFY*, *AtSOC1* and *AtAP1* were normalized to that of *ACTIN2*. Data are mean ±SD, n=3.


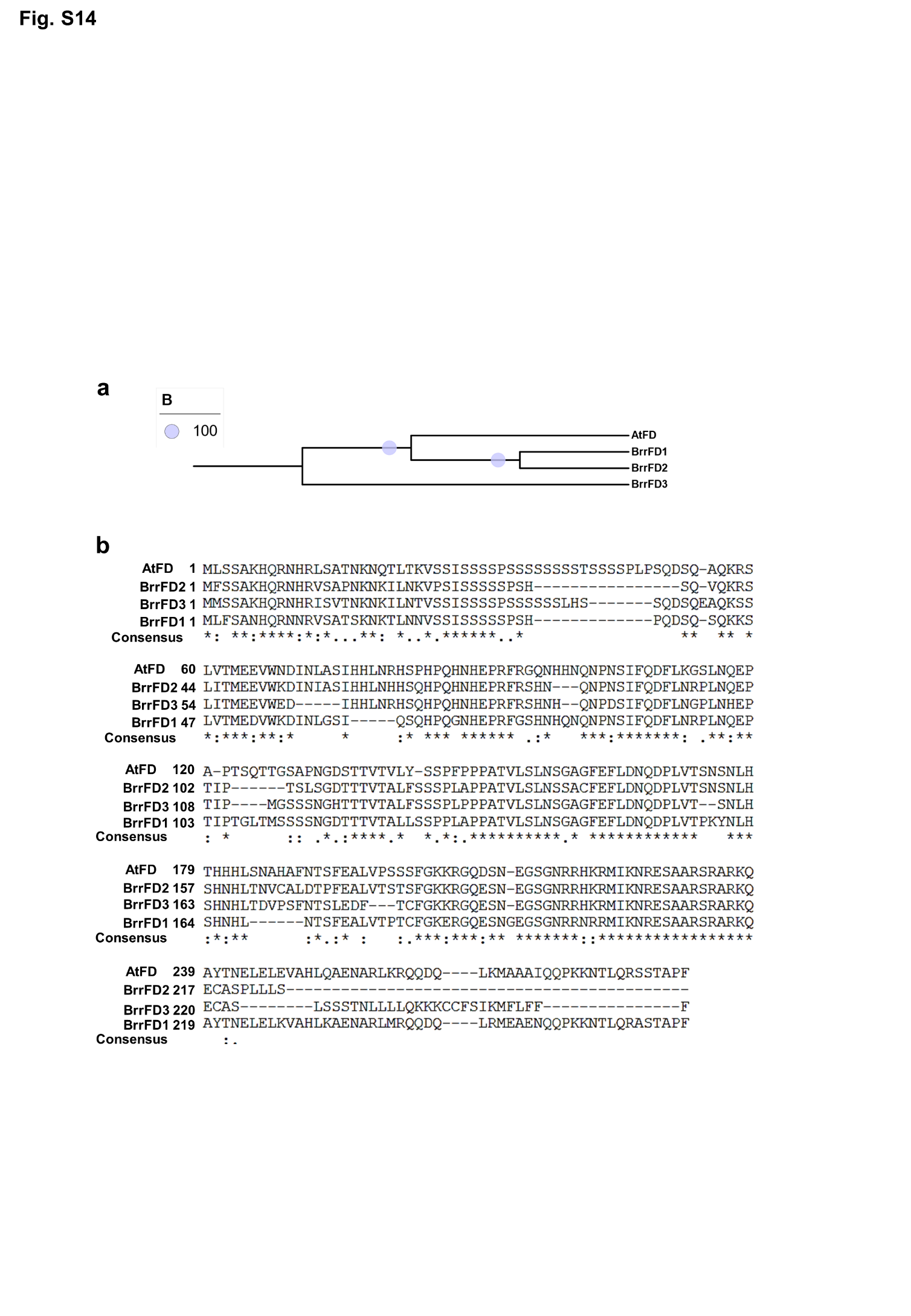


**Fig. S14. Sequence analysis of BrrFD paralogues in *B. rapa* ssp*. rapa*.** (a) Phylogenetic tree of AtFD and three BrrFD proteins using TreeBeST v.0.2.0 software. (b) Multiple sequence alignments based on full-length protein sequences of AtFD and three BrrFD paralogues.


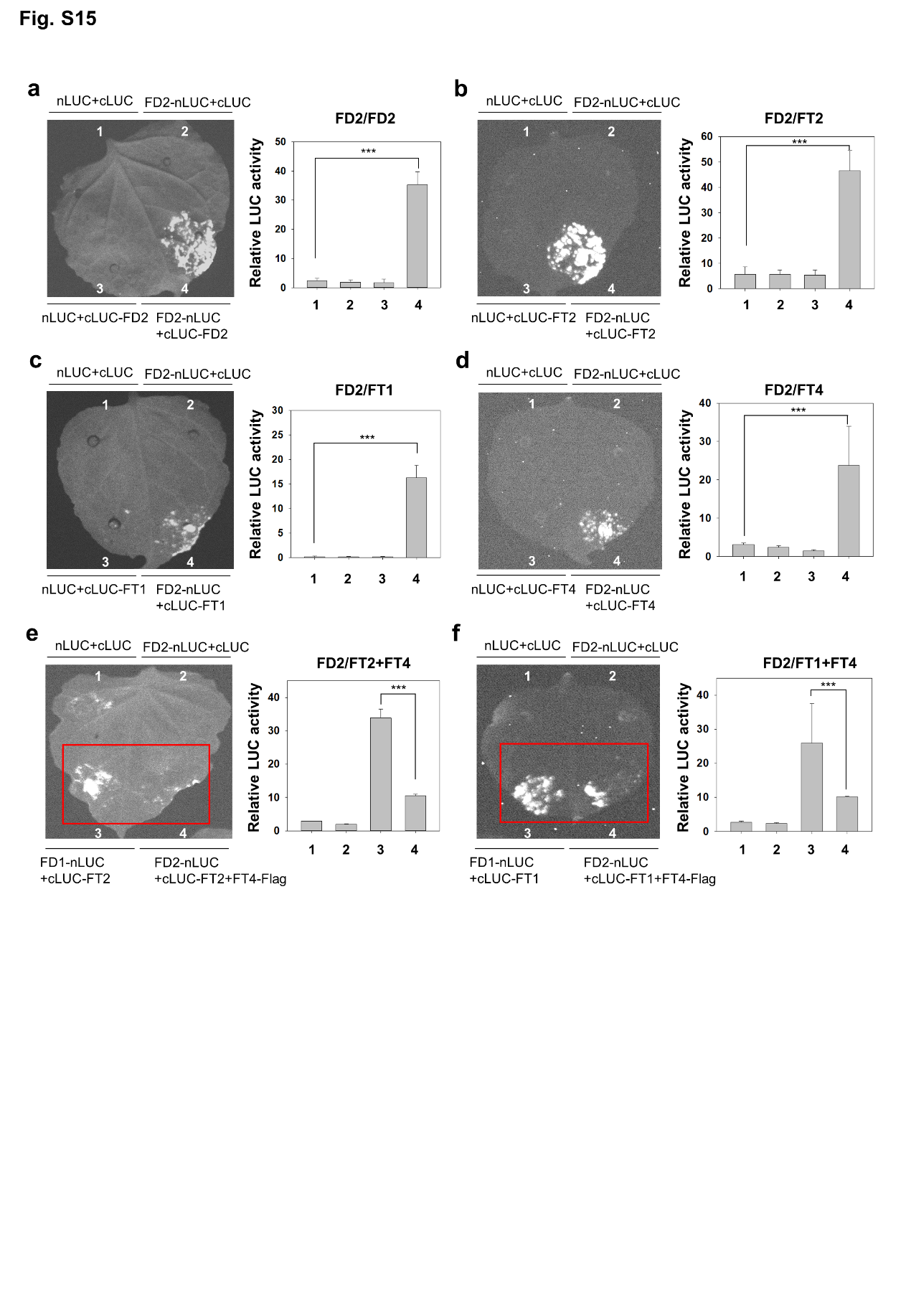


**Fig. S15. LCI assays between BrrFT1, 2, 4 and BrrFD2 in *N. benthamiana* leaves.** (a) LCI assays between BrrFD2 and BrrFD2. (b) LCI assays between BrrFT2 and BrrFD2. (c) LCI assays between BrrFT1 and BrrFD2. (d) LCI assays between BrrFT4 and BrrFD2. (e) LCI assays to identify the relationship of BrrFT2 and BrrFT4 on interacting with BrrFD2. (f) LCI assays to identify the relationship of BrrFT1 and BrrFT4 on interacting with BrrFD2. The relative LUC activity is displayed next to the image. Data are the mean ± SD. n = 5. Statistical analyses were performed using ordinary one-way ANOVA followed by Turkey’s multiple comparisons test compared to that of the negative control, ***, P<0.001.
